# Machine learning-based early screening of mild cognitive impairment using nutrition-related biomarkers and functional indicators

**DOI:** 10.3389/fnagi.2025.1641690

**Published:** 2025-12-05

**Authors:** Ling Yuan, Yao Zhang, Yiwen Wu, Anke Zhang, He Bai, Mengyao He, Zhaoxin Wang, Liqiang Zheng

**Affiliations:** 1 School of Public Health, Shanghai Jiao Tong University School of Medicine, Shanghai, China; 2 Department of Endocrinology, Shengjing Hospital of China Medical University, Shenyang, China; 3 Department of Neurosurgery, Second Affiliated Hospital, School of Medicine, Zhejiang University, Hangzhou, China; 4 School of Public Health, China Medical University, Shenyang, China; 5 School of Public Health, Hainan Medical University, Haikou, China; 6 Clinical Research Centre, The International Peace Maternity and Child Health Hospital, Shanghai Jiao Tong University School of Medicine, Shanghai, China; 7 Ministry of Education-Shanghai Key Laboratory of Children's Environmental Health, Xinhua Hospital Affiliated to Shanghai Jiao Tong University School of Medicine, Shanghai, China

**Keywords:** mild cognitive impairment, machine learning, SHAP, TMAO, choline, carnitine, betaine, sleep quality

## Abstract

**Objectives:**

Mild cognitive impairment (MCI), an early stage of cognitive decline preceding dementia, poses a growing public health concern, especially in aging populations. Early identification of individuals at risk is essential for implementing timely interventions to delay or prevent progression to dementia. Nutritional factors and related biomarkers have emerged as promising targets for developing convenient, scalable screening strategies, particularly in resource-limited rural settings. This study aimed to develop and validate a machine learning (ML) model that integrates diet-related metabolites, physical examination indicators, lifestyle behaviors, and sleep quality to predict MCI risk and to evaluate the biological and predictive relevance of trimethylamine N-oxide (TMAO) and its dietary precursors among older adults in rural China.

**Methods:**

Data were derived from a large-scale epidemiological survey in Fuxin County, Liaoning Province, including 907 participants, of whom 270 were classified as MCI based on the Montreal Cognitive Assessment-Basic. Seven ML models were trained and evaluated using accuracy, sensitivity, and the area under the receiver operating characteristic curve (AUC). The best model’s predictors were interpreted using Shapley Additive Explanation (SHAP) values.

**Results:**

The random forest model showed the bestperformance (AUC = 0.74, 95% CI: 0.677–0.801; sensitivity = 0.72). SHAP analysis identified age, choline, carnitine, betaine, TMAO, daily intake of fruit and vegetables, body mass index, hip circumference, and daytime dysfunction as key predictors.

**Conclusion:**

TMAO-related metabolites consistently contributed positive SHAP effects, suggesting biologically relevant links between dietary metabolism and early cognitive decline. This interpretable ML framework offers a feasible, sensitive, and biologically informed approach for early MCI screening and supports the integration of nutritional biomarkers into cognitive health surveillance.

## 1 Introduction

Dementia has emerged as a predominant contributor to disability in the global population aged 65 years and older (GBD 2019 Dementia Forecasting Collaborators, 2022), with China bearing a significant burden, as evidenced by the estimated 15.07 million dementia cases among individuals aged 60 and above, representing approximately 25.5% of the global dementia population (Jia et al., 2020). Given the absence of definitive pharmacological interventions for dementia (Tahir et al., 2021), emphasis has shifted toward early detection and preventive strategies. Mild cognitive impairment (MCI) is a transitional stage between the expected cognitive decline of normal aging and the more serious decline of dementia, also a critical stage in the early prevention and control of dementia. It is characterized by noticeable changes in cognitive functions, such as memory and thinking skills, which are greater than normal for a person’s age and education level but not severe enough to interfere significantly with daily life. Epidemiological data reveal that individuals with MCI demonstrate a tenfold increasing annual progression rate to dementia compared to cognitively intact individuals (Song et al., 2021; Alzheimer’s Disease Facts and Figures, 2023; Petersen et al., 2018). Therefore, widespread and effective screening for MCI is essential for the early prevention and control of dementia, playing a critical role in enabling timely interventions and improving long-term health outcomes.

In China, the proportion of people aged 60 and above with cognitive impairment has been rising and reached 18.70% in 2020 (Ren et al., 2022). In 2024, 15 departments including the National Health Commission jointly issued the National Action Plan for Addressing Dementia in Older Adults (2024–2030), which explicitly proposes advancing preliminary cognitive function screening for older adults in communities by integrating services such as health management and health check-ups (Commission NH, 2017). However, healthcare-seeking rate for cognitive impairment remains low, while the rate of underdiagnosis is markedly high. This is attributable, in part, to the insufficiency of specialized medical resources. For instance, fewer than 2% of neurologists in general tertiary hospitals possess the capacity to assess and manage dementia, and pronounced disparities in healthcare infrastructure between urban and rural regions further hinder timely identification (Jia et al., 2016). Consequently, a substantial proportion of individuals with early-stage cognitive impairment remain undiagnosed and do not receive appropriate referrals (Partridge et al., 2014). Additionally, limited public awareness and the stigma associated with cognitive disorders contribute to delays in seeking medical attention, resulting in missed opportunities for early diagnosis and intervention (Isaacson and Saif, 2020). While traditional cognitive screening tools, such as Mini-mental State Examination (MMSE) and Montreal Cognitive Assessment (MoCA) are gold standards in clinical settings, we aim to develop an algorithm that allows for identifying individuals at MCI risk using a convenient and rapid approach, without relying on complex questionnaires.

While established risk factors such as familial dementia history, hypertension, and diabetes mellitus contribute to MCI pathogenesis (Livingston et al., 2020; Hendriks et al., 2024), these account for only a proportion of cases, necessitating further exploration of additional etiological factors influencing MCI development. Given its potential to progress to dementia, particularly Alzheimer’s disease (AD), understanding the risk factors and mechanisms underlying MCI is crucial for early intervention and prevention strategies.

Recent research has highlighted the role of gut microbiota-derived metabolites in cognitive health. Trimethylamine N-oxide (TMAO), a gut microbiota-derived metabolite, has emerged as a significant biomarker in cardiovascular disease and, more recently, cognitive impairment (Zeisel and Warrier, 2017; Zhou et al., 2023; Xu et al., 2022). Elevated levels of TMAO have been associated with an increased risk of cardiovascular events, and there is growing evidence suggesting a link between TMAO and cognitive decline. This biologically active compound originates from the microbial metabolism of specific dietary substrates, including choline (and its derivatives), carnitine, and betaine (Zhu et al., 2014). The metabolic cascade involves initial conversion of these precursors to trimethylamine (TMA) by intestinal microbiota, followed by portal system-mediated hepatic transport where flavin-containing monooxygenase 3 (FMO3) catalyzes the final oxidation step to produce circulating TMAO (Liu et al., 2023). Emerging evidence indicates that increased circulating TMAO concentrations may compromise neurological homeostasis through multiple pathways, including blood–brain barrier disruption, induction of neuroinflammatory cascades, and activation of immune-mediated processes (Zhu et al., 2014). Nevertheless, current research presents conflicting evidence regarding these mechanisms, with substantial knowledge gaps remaining in understanding TMAO’s neuropathological effects (Liu et al., 2023; Casagrande et al., 2022; Song and Yu, 2019; Li et al., 2022).

Sleep quality is another critical factor that has been linked to cognitive impairment (Casagrande et al., 2022). Poor sleep quality, characterized by difficulties in falling asleep, staying asleep, or experiencing restorative sleep, has been associated with an increased risk of MCI and dementia (Song and Yu, 2019). Sleep plays a vital role in cognitive processes, including memory consolidation and synaptic plasticity. Disruptions in sleep patterns can lead to the accumulation of neurotoxic proteins, such as beta-amyloid, which are implicated in the development of Alzheimer’s disease. Moreover, sleep disturbances can exacerbate other risk factors for cognitive decline, such as cardiovascular disease and depression (Li et al., 2022). The exact mechanisms by which sleep quality influences cognitive function are not fully understood, but it is hypothesized that sleep quality may promote neuroinflammation, oxidative stress, and endothelial dysfunction, all of which are implicated in the pathogenesis of MCI and dementia. Machine learning (ML) has emerged as a powerful tool in medical research for the prediction of disease outcome. ML algorithms can analyze complex, high-dimensional data and identify patterns that may not be apparent through traditional statistical methods (Qi et al., 2025; Liu et al., 2024; Zhong et al., 2023). High-dimensional data analysis has demonstrated strong capability in extracting key features essential for the identification of health-related conditions (Jing et al., 2023). Given the multifactorial etiology of MCI, developing identification models that integrate multiple risk factors holds substantial clinical application potential.

In this study, we used data from a large-scale epidemiological study in the rural areas of Fuxin County, Liaoning Province, China, collected in 2019, encompassing a total of 1,294 participants. Cognitive impairment was classified based on the Chinese version of the Montreal Cognitive Assessment-Basic (MoCA-BC) scores, with a cutoff point changed by education years. We included serum TMAO and its precursors, lifestyle behaviors, disease history, general physical examination and Pittsburgh sleep quality index (PSQI) scores in the ML models for training, aiming to predict the occurrence of MCI, and to evaluate the biological and predictive relevance of TMAO-related metabolites within a multimodal framework. To optimize analytical rigor, we implemented a comprehensive benchmarking framework for model evaluation and incorporated Shapley Additive Explanation (SHAP) values to improve model interpretability, quantifying both the discriminative importance and potential biological implications of each feature in MCI risk identification. This research not only provides crucial population-based evidence from rural China on the life style and MCI relationship but also highlights TMAO and its dietary precursors as biologically meaningful nutritional biomarkers that may help guide early preventive strategies through dietary modulation.

## 2 Materials and methods

### 2.1 Participants

The data for this study were obtained from a large-scale epidemiological survey conducted in rural areas of Fuxin County, Liaoning Province, China. The baseline assessment took place between June and August 2019. Details regarding the selection of villages, questionnaire administration, physical examinations, and other study procedures have been previously described in our published literature (Huang et al., 2024). Participants were eligible if they: (1) were aged 35 years or older, (2) had resided in the study area for at least 5 years, and (3) provided written informed consent. Exclusion criteria included pregnancy, severe hepatic or renal dysfunction, and unwillingness to participate. A total of 4,689 individuals were enrolled. Written informed consent was obtained from all participants. The study was approved by the Human Experimentation Committee of China Medical University [No. (2018)083].

**Figure 1:**
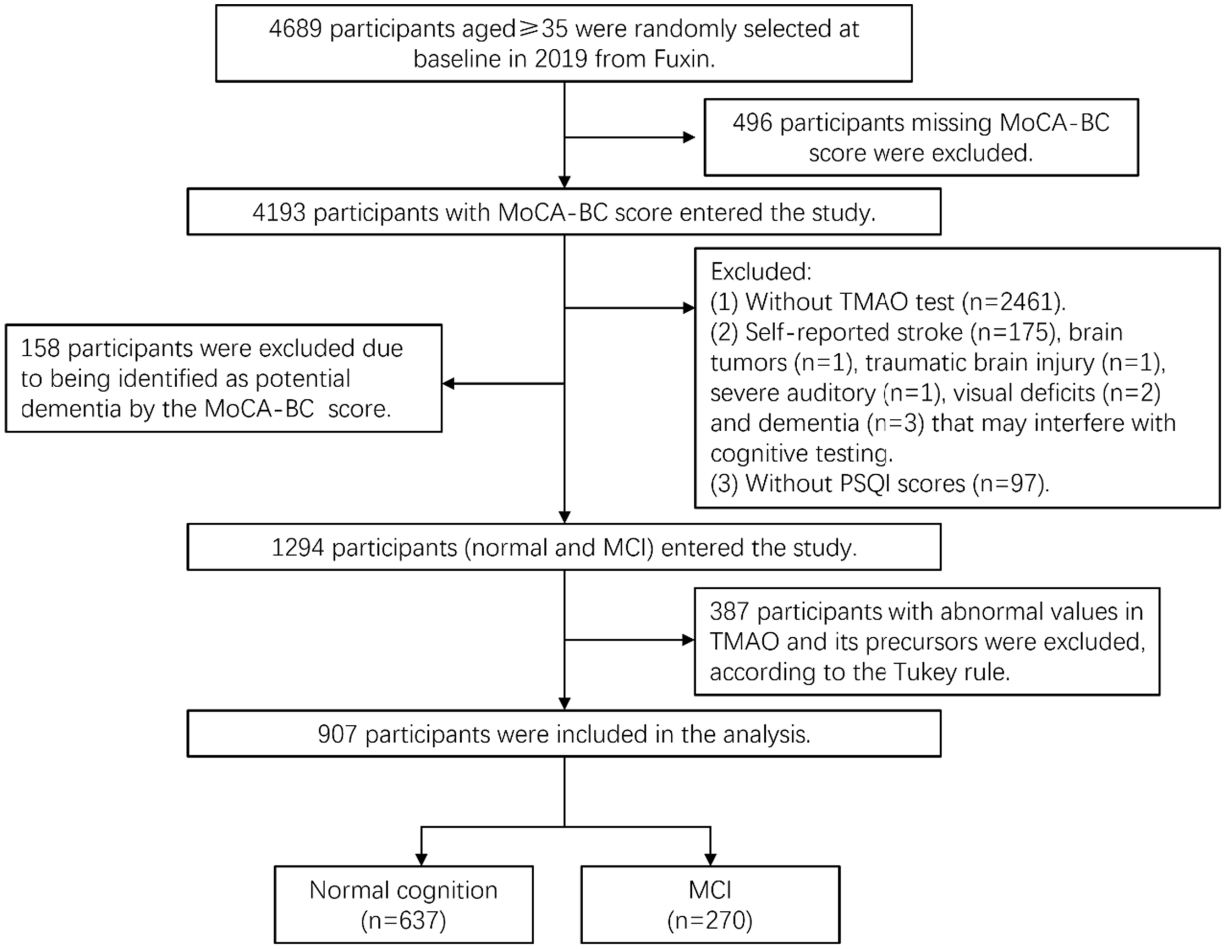
Flow diagram for the inclusion/exclusion of participants. TMAO, trimethylamine N-oxide; MCI, mild cognitive impairment; MoCA-BC, the Chinese version of the Montreal Cognitive Assessment-Basic.

The inclusion and exclusion criteria for this study are illustrated in Figure 1. Among the initial 4,689 participants, 496 participants were excluded due to without MoCA-BC scores, and 2,461 participants without measurements of TMAO and its precursors were excluded. Additionally, 183 individuals were excluded based on self-reported histories of stroke, Alzheimer’s disease, brain tumors, traumatic brain injury, severe auditory, and visual or motor deficits that may interfere with cognitive testing, while 97 participants without PSQI scores were excluded as well. Meanwhile, 158 participants were suspected dementia, according to MoCA-BC scores were also removed. According to the Tukey rule (Rykov et al., 2021), 387 participants with abnormal values in TMAO and its precursors were excluded. A total of 907participants were included in this study (Figure 1) and randomly divided into a training set (*n* = 634, 70%) and an internal test set (*n* = 273, 30%).

### 2.2 Quantification of serum TMAO and its precursors

Serum concentrations of TMAO and its precursors were quantified using high-performance liquid chromatography–tandem mass spectrometry (HPLC-MS/MS) (Shimadzu, Japan), with fasting venous blood samples collected from participants, centrifuged at 3000 rpm for 10 min, and stored at −80 °C after extracting a 500 μL aliquot of the supernatant. For analysis, 10 μL of serum was diluted 50-fold with a standard solution, vortexed for 3 min, and centrifuged at 15,000 rpm for 15 min at 4 °C, followed by filtration through a 0.22 μm hydrophobic nylon membrane and transfer of 100 μL of the filtered serum into a 1.5 mL sample vial. Chromatographic separation was performed using an ACQUITY UPLC HSS T3 column (1.7 μm, 2.1 mm × 100 mm; Waters, United States) with an injection volume of 2 μL and a column temperature of 40 °C, employing a gradient elution protocol with mobile phases consisting of Phase A (10 mmol/L ammonium formate, 0.1% formic acid, and acetonitrile-water in a 9:1 ratio) and Phase B (5 mmol/L ammonium formate, 0.1% formic acid, and acetonitrile-water in a 1:1 ratio). The gradient program included maintaining Phase B at 10% from 0.0 to 2.0 min, linearly increasing it to 45% by 6.0 min, further increasing it to 100% by 6.1 min, holding it at 100% until 8.1 min, decreasing it back to 10% by 8.2 min, and maintaining it at 10% until 10.0 min, with a flow rate of 0.4 mL/min (split ratio 5:3) and a total runtime of 10 min per sample, including a 2.5-min equilibration period. This method ensured high sensitivity and specificity for quantifying TMAO and its precursors, enabling accurate assessment of their serum levels in the study population.

To ensure precise quantification of metabolites, reference standards were employed to establish chromatographic retention times and optimize mass spectrometry parameters. The analysis was conducted using an Electrospray Ionization Source in positive ion mode, with an impact voltage set at 20 eV. Multiple Reaction Monitoring (MRM) was utilized under optimized operating conditions, including a drying gas flow rate of 3 L/min, an atomizer pressure of 50 psi, a drying temperature of 350 °C, and a capillary voltage of 3,500 V. The method demonstrated a linear detection range of 0.16 to 20.00 μmol/L, with an exceptional correlation coefficient (r^2^ = 0.999), and achieved recovery rates for the metabolites ranging from 90.2 to 102.1%, confirming the accuracy and reliability of the analytical approach.

### 2.3 MoCA-BC test and diagnosis of MCI

The MoCA-BC serves as a screening tool for MCI in elderly Chinese individuals with diverse educational backgrounds. This assessment evaluates nine cognitive domains—executive function, language, orientation, calculation, conceptual thinking, memory, visual perception, attention, and concentration—with a total score of 30. Classification into normal cognition (NC), MCI, or potential dementia is based on educational level: (1) for individuals with ≤6 years of education, NC corresponds to scores of 19–30, MCI to 13–18, and potential dementia to 0–12; (2) for those with 7–12 years, NC ranges from 22 to 30, MCI from 15 to 21, and potential dementia from 0 to 14; (3) for individuals with >12 years, NC falls within 24–30, MCI within 16–23, and potential dementia within 0–15. In this cross-sectional study, the MoCA-BC was used to identify NC, MCI, and potential dementia, with individuals classified as having potential dementia excluded.

### 2.4 Sleep quality

Sleep was assessed using PSQI including seven characterizes: subjective sleep quality, sleep onset latency, sleep duration, sleep efficiency, disturbance, hypnotic drug use, and daytime disfunction (Buysse et al., 1989). Each sleep domain is rated from 0 to 3, and the total sleep score derived from seven combined components spans from 0 to 21, where higher scores reflect worse sleep quality (Hu et al., 2025). A total score exceeding 5 was classified as indicative of poor overall sleep (Nevels et al., 2023).

### 2.5 Predictors

Data on demographic predictors (age, sex family members, education, marital status, ethnicity, and income), history of disease (diabetes, hypertension, coronary heart disease), lifestyle predictors (weight management, smoking, drinking, home passive smoking, tea, physical labour level, moderate-intensity exercise), and habitual dietary intakes (daily intake of fruit and vegetables, weekly intake of vegetables, fish, sugar, beverage, and cereal, daily salt intake, and daily oil intake) were collected from a standardized face-to-face questionnaires. Education was divided into three levels: primary school or below, middle school, and high school or above. Ethnicities were divided into three groups with Han, Mongolian, and others, as the local is a Mongolian autonomous county with a relatively high proportion and the living habits differ from those of Han. Income levels were classified as low (<10,000 yuan, below the local average) and high (≥10,000 yuan, abovethe local average). Weight management refers to a series of behaviors aimed at weight control in a planned and active manner within the past year, and the duration should not be less than 1 week. The assessment of physical labor intensity referred to the National Standard of the People’s Republic of China (GB3869-1997) (Chen et al., 2025), which was formerly divided into four groups: (1) sitting or standing posture mainly using upper arm strength; (2) continuous arm work or/and with legs and torso; (3) workload with arms and torso; (4) extremely intense activities. We combined 3 and 4 and recategorized them as low, moderate, and high. Current smokere was classified as consuming at least one cigarette per day for a minimum of 6 months, while current drinker was classified based on the Dietary Guidelines for Chinese Residents, with drinkers identified as those consuming alcohol at least three times per week for six consecutive months.

Height and weight were measured using a calibrated domestic instrument (Sitai Corporation, China), and Body mass index (BMI) was calculated as weight (kg) divided by height (m^2^). Waist and hip circumferences were measured using a tape, and waist-hip ratio (WHR) was calculated as waist circumference divided by hip circumference. Blood pressure was measured with the HEM-8102A/K electronic monitor (Omron Corporation, Japan), with three readings taken at intervals of over 1 min, and systolic (SBP) and diastolic blood pressure (DBP) calculated as the average of these measurements. Fasting blood glucose (GLU) was determined using the glucose oxidase method, alanine aminotransferase (ALT) by colorimetry, total cholesterol (TC), low-density lipoprotein cholesterol (LDL-C), high-density lipoprotein cholesterol (HDL-C), and creatinine through enzymatic colorimetry, and uric acid was measured by uricase-peroxidase method. The above blood biochemical analyses performed using the Cobas 8,000 C701 automated analyzer (Roche, Switzerland), and converted into categorical variables according to the reference interval published by the National Health Commision of the People’s Republic of China (China NHCotPsRo, 2018).

### 2.6 Data preprocessing

The data preprocessing phase addressed missing values using multiple imputation by chained equations (MICE). This method was applied to impute missing data for individuals lacking information on lifestyle and habitual habitual dietary intake, adjusting for all measured variables potentially associated with the missingness (Carson et al., 2023).

Before imputation, the extent and pattern of missing data were summarized for each variable, including the number and proportion of missing observations as well as descriptive statistics (mean, SD, median, minimum, maximum, skewness, and kurtosis) (Supplementary Table S1).

To assess the reliability of the imputation, we compared the key summary statistics (mean, median, and SD) of each variable before and after imputation and visualized the distributions of continuous variables using histograms. These comparisons indicated consistency between pre- and post-imputation data, suggesting that the MICE procedure preserved the original data characteristics and did not introduce noticeable distortion.

### 2.7 Feature selection

High-dimensional data with many predictors are susceptible to overfitting, making effective variable selection essential. The Boruta algorithm, which leverages random forest principles, excels at identifying important predictors by evaluating their relationship with the outcome variable. Thus, we applied this technique to determine the most relevant predictors from a set of 52 variables (16 continuous variables and 36 categorical ones). Acceptable variables were subsequently integrated into the machine learning algorithm.

The Boruta algorithm identifies influential variables by repeatedly training random forest models on bootstrapped datasets and measuring variable importance using indicators like mean decrease impurity (Saleem et al., 2024). To assess whether a feature contributes meaningfully, Boruta introduces “shadow features,” which are randomized versions of the original variables. These shadow features are merged with the actual variables to build an extended dataset, where each feature’s relevance is evaluated by comparing its Z-score to those of its shadow counterparts. In brief, if a feature’s Z-score consistently surpasses the highest Z-score among the shadow features across iterations, it is marked as “confirmed” (green), signifying statistical relevance. Features with Z-scores similar to shadow features are labeled “tentative” (yellow), requiring additional assessment. In contrast, those unable to outperform their shadow equivalents are considered “unimportant” (red) and excluded. This technique is especially suitable for nonlinear, high-dimensional data, is tolerant to noise and missing values, and enhances prediction when used with diverse machine learning frameworks, all without assuming specific data distributions (Yan et al., 2024).

To counterbalance the tree-model preference of Boruta, we further adopted Least Absolute Shrinkage and Selection Operator (LASSO) regression and Support Vector Machine–Recursive Feature Elimination (SVM-RFE), both capable of selecting informative predictors through different regularization and margin-based mechanisms. Additionally, a no feature selection condition was tested as a baseline reference. The results for different feature selection methods are presented in Supplementary Table S2. These four approaches—Boruta, LASSO, SVM-RFE no selection,—allowed a comprehensive comparison of feature selection effects on model performance and consistency across algorithms (Supplementary Tables S3–S6).

### 2.8 Model construction, evaluation, and interpretation

The selection of ML models was driven by a desire to compare different algorithms’ performance and assess their robustness in predicting MCI in this study. We chose 7 widely used algorithms due to their diverse classification strategies and strengths in processing various data structures. Random forest (RF), Gradient Boosting Machine (GBM), and Extreme Gradient Boosting (XGBoost) are ensemble-based approaches, well-suited for managing complex, high-dimensional inputs and identifying nonlinear patterns. Logistic regression (LR) and support vector machines (SVM) are traditional binary classifiers, with SVM particularly effective in managing datasets with many features. k-Nearest Neighbors (KNN) is a distance-based non-parametric method that classifies data based on the majority of nearby neighbors, working well with small datasets. Decision tree (DT) is a single-tree model known for its fast speed and clear interpretation, and is often used as the base for ensemble methods.

The construction of each model followed a systematic approach to ensure rigorous evaluation and validation. The data was randomly divided into training set (634/907, 70%) and test set (273/907, 30%). Each algorithm was trained under four feature selection conditions (None, Boruta, LASSO, and SVM-RFE) to ensure comparability.

A random search strategy with up to 1,000 evaluations was employed within each learner-specific parameter space, using AUC as the optimization criterion. The tuning process selected the parameter configuration that maximized the five-fold cross-validated AUC. Subsequently, the best-performing hyperparameters were applied to retrain each model on the full training data, followed by independent testing on the held-out dataset. This approach ensured reproducibility and prevented data leakage between model optimization and evaluation stages. The hyperparameter variables selected for optimization and their corresponding final optimized values for each learner are presented in Supplementary Tables S2–S6.

SHAP (Shapley Additive Explanations) is a technique that helps interpret machine learning model outputs by assigning each feature a contribution score, clarifying how individual predictors influence the model’s results. In this study, SHAP was applied to explain the model predicting cognitive impairment in rural adults (Guan et al., 2024). By breaking down the prediction into feature-level contributions, SHAP provides a clear and interpretable way to link the model’s decisions to specific risk factors.

### 2.9 Evaluation metrics

In this study, the performance of seven ML models was evaluate using six metrics: accuracy, precision, recall, F1 score, the area under the receiver operator characteristic curve (AUC-ROC), and the area under the recision-recall (PR) curve. The AUC-ROC was prioritized as the primary metric for model selection, given its robustness in evaluating classification performance. Other metrics served as supplementary tools to provide a comprehensive assessment of model behavior.

### 2.10 Statistical analysis

Continuous variables were summarized as mean ± standard deviation (SD) or median [interquartile range, IQR], while categorical variables were expressed as frequencies (percentages). The chi-square (*χ*^2^) test, Fisher’s exact test, and Wilcoxon rank-sum test were used for categorical variables. Student’s t test and Mann–Whitney U test were used to compare continuous differences.

All statistical analyses are conducted using R software (version 4.3.1). Key R packages utilized in this study includes mice, tidyverse, ggplot2, mlr3, mlr3viz, mlr3learners, mlr3verse, mlr3tuning, data.table, mlr3extralearners, Boruta, DALEX, kernelshap, and shapviz. Statistical tests were two-sided and a *p*-value <0.05 was considered statistically significant.

## 3 Result

### 3.1 Characteristics of the features

Table 1 details the baseline characteristics of the 907 participants included in this study, stratified into normal cognition (NC, *n* = 637) and MCI (*n* = 270) cohorts. Overall, the mean age was 58.9 ± 9.4 years and over half of the participants were females (69.90%). Comparative analyses between the MCI and NC groups revealed significant differences in several key variables. Specifically, the MCI group had more olders (58.52%), fewer females (61.11%), smaller hip circumference [94.00 (90.00, 99.00)], elevated SBP [133.33 (119.08, 146.92)], and more smokers (37.41%). Notably, levels of TMAO and its precursors, including choline, betaine, and carnitine, were consistently elevated in the MCI group (*p* < 0.05). Sleep quality analysis revealed sleep onset latency (*p* = 0.024), daytime disfunction (*p* = 0.004) were significantly different in NC and MCI groups. These findings highlight distinct demographic and biochemical profiles associated with MCI, underscoring the potential role of metabolic factors in cognitive impairment.

**Table 1:** Clinical baseline of participates.

Characteristics	Total *N* = 907	NC *N* = 637	MCI *N* = 270	*p*-value
Age, ≥ 60y	450 (49.61)	292 (45.84)	158 (58.52)	**0.001**
Male, *n* (%)	273 (30.10)	168 (26.37)	105 (38.89)	**<0.001**
Education level, *n* (%)				0.231^a^
Primary school or below	344 (37.93)	237 (37.21)	107 (39.63)	
Junior high school	409 (45.09)	283 (44.43)	126 (46.67)	
Tertiary high school or higher	154 (16.98)	117 (18.37)	37 (13.70)	
Marital status, *n* (%)				
Independent	817 (90.08)	571 (89.64)	246 (91.11)	0.574
Married	76 (8.38)	57 (8.95)	19 (7.04)	
Widowhood	14 (1.54)	9 (1.41)	5 (1.85)	
Ethnicity, *n* (%)				
Han	568 (62.62)	398 (62.48)	170 (62.96)	0.718
Mongolian	305 (33.63)	213 (33.44)	92 (34.07)	
Others	34 (3.75)	26 (4.08)	8 (2.96)	
Income, <10,000 Yuan, *n* (%)	635 (70.01)	445 (69.86)	190 (70.37)	0.941
Family members, person	2.00 [2.00, 4.00]	3.00 [2.00, 4.00]	2.00 [2.00, 4.00]	0.502
Diabetes, *n* (%)	63 (6.95)	43 (6.75)	20 (7.41)	0.831
Hypertension, *n* (%)	236 (26.02)	164 (25.75)	72 (26.67)	0.837
CHD, *n* (%)	135 (14.88)	88 (13.81)	47 (17.41)	0.198
High, cm	160.00 [155.00, 165.00]	160.00 [155.00, 165.00]	159.50 [155.00, 165.00]	0.651
Weight, kg	63.00 [56.00, 71.00]	64.00 [56.00, 71.50]	62.00 [56.00, 69.00]	0.106
BMI, kg/m^2^	24.67 [22.15, 27.19]	24.80 [22.19, 27.34]	24.38 [22.04, 26.49]	0.117
Waist circumference, cm	84.00 [77.00, 91.00]	85.00 [77.00, 91.00]	84.00 [77.00, 90.00]	0.616
Hip circumference, cm	95.00 [90.00, 100.00]	95.00 [91.00, 100.00]	94.00 [90.00, 99.00]	**0.043**
WHR	0.88 (0.06)	0.88 (0.06)	0.89 (0.06)	0.133
SBP, mmHg	131.00 [117.33, 144.33]	130.00 [117.00, 143.33]	133.33 [119.08, 146.92]	**0.019**
DBP, mmHg	79.00 [72.00, 86.67]	78.67 [71.67, 86.67]	79.83 [73.00, 87.25]	0.117
Heart rate, bpm	73.67 [68.00, 80.67]	73.33 [67.33, 80.33]	75.00 [68.67, 80.67]	0.298
Daily salt intake, g	16.67 [12.50, 25.00]	16.67 [12.50, 25.00]	16.67 [13.33, 25.00]	**0.012**
Daily oil intake, ml	166.67 [83.33, 166.67]	166.67 [83.33, 166.67]	166.67 [111.11, 166.67]	0.309
Weight management, *n* (%)	53 (5.84)	41 (6.44)	12 (4.44)	0.31
Current smoker, *n* (%)	291 (32.08)	190 (29.83)	101 (37.41)	**0.031**
Current drinker, *n* (%)	185 (20.40)	119 (18.68)	66 (24.44)	0.06
Home passive smoker, *n* (%)	322 (35.50)	224 (35.16)	98 (36.30)	0.803
Tea, *n* (%)	485 (53.47)	339 (53.22)	146 (54.07)	0.87
Daily intake of fruit and vegetables, *n* (%)				0.549
<250 g	36 (3.97)	26 (4.08)	10 (3.70)	
250 g-500 g	426 (46.97)	306 (48.04)	120 (44.44)	
>500 g	445 (49.06)	305 (47.88)	140 (51.85)	
Weekly intake of fish, *n* (%)				**0.042**
<100 g	577 (63.62)	394 (61.85)	183 (67.78)	
100 g-250 g	295 (32.52)	222 (34.85)	73 (27.04)	
>250 g	35 (3.86)	21 (3.30)	14 (5.19)	
Weekly intake of sugar, *n* (%)				0.156
<50 g	596 (65.71)	420 (65.93)	176 (65.19)	
50 g-100 g	280 (30.87)	200 (31.40)	80 (29.63)	
>100 g	31 (3.42)	17 (2.67)	14 (5.19)	
Weekly intake of beverage, <500 mL, *n* (%)	868 (95.70)	605 (94.98)	263 (97.41)	0.141
Weekly intake of cereal, *n* (%)				**0.007**
<100 g	167 (18.41)	117 (18.37)	50 (18.52)	
100 g-250 g	190 (20.95)	145 (22.76)	45 (16.67)	
250 g-1 kg	257 (28.34)	190 (29.83)	67 (24.81)	
>1 kg	293 (32.30)	185 (29.04)	108 (40.00)	
Physical labour level, *n* (%)				0.373
Moderate	250 (27.56)	173 (27.16)	77 (28.52)	
Low	621 (68.47)	435 (68.29)	186 (68.89)	
High	36 (3.97)	29 (4.55)	7 (2.59)	
Moderate intensity exercise, weekly times, *n* (%)				0.913
<3	696 (76.74)	490 (76.92)	206 (76.30)	
3–4	53 (5.84)	38 (5.97)	15 (5.56)	
>4	158 (17.42)	109 (17.11)	49 (18.15)	
TMAO, μmol/L	3.56 [2.02, 5.84]	3.32 [2.01, 5.64]	4.11 [2.10, 6.63]	**0.032**
Choline, μmol/L	165.03 [120.21, 218.33]	157.35 [112.09, 208.19]	185.44 [147.30, 235.84]	**<0.001**
Betaine, μmol/L	94.19 [72.33, 127.99]	91.56 [70.16, 119.68]	104.77 [76.99, 141.03]	**<0.001**
Carnitine, μmol/L	48.57 [39.13, 61.25]	47.33 [38.22, 57.94]	51.86 [41.04, 67.88]	**<0.001**
ALT, abnormal, *n* (%)	39 (4.30)	33 (5.18)	6 (2.22)	0.067
GLU, abnormal, *n* (%)	213 (23.48)	144 (22.61)	69 (25.56)	0.383
TCHOL, abnormal, *n* (%)	419 (46.20)	287 (45.05)	132 (48.89)	0.324
HDL, abnormal, *n* (%)	251 (27.67)	180 (28.26)	71 (26.30)	0.601
LDL, abnormal, *n* (%)	401 (44.21)	280 (43.96)	121 (44.81)	0.869
Creatinine, abnormal, *n* (%)	22 (2.43)	16 (2.51)	6 (2.22)	0.982
Uric acid, abnormal, *n* (%)	52 (5.73)	37 (5.81)	15 (5.56)	1
Subjective sleep quality, *n* (%)				0.986
Very good (0 score)	320 (35.28)	224 (35.16)	96 (35.56)	
Fairly good (1 score)	396 (43.66)	278 (43.64)	118 (43.70)	
Fairly bad (2 score)	153 (16.87)	109 (17.11)	44 (16.30)	
Very bad (3 score)	38 (4.19)	26 (4.08)	12 (4.44)	
Sleep onset latency, *n* (%)				**0.024**
Fast (0 score)	408 (44.98)	307 (48.19)	101 (37.41)	
Fairly slow (1 score)	258 (28.45)	167 (26.22)	91 (33.70)	
Slow (2 score)	137 (15.10)	93 (14.60)	44 (16.30)	
Very slow (3 score)	104 (11.47)	70 (10.99)	34 (12.59)	
Sleep duration, *n* (%)				0.34
>7 h (0 score)	232 (25.58)	159 (24.96)	73 (27.04)	
6–7 h (1 score)	347 (38.26)	255 (40.03)	92 (34.07)	
5–6 h (2 score)	227 (25.03)	157 (24.65)	70 (25.93)	
<5 h (3 score)	101 (11.14)	66 (10.36)	35 (12.96)	
Sleep efficiency, *n* (%)				0.087
>85% (0 score)	773 (85.23)	547 (85.87)	226 (83.70)	
75–84% (1 score)	63 (6.95)	43 (6.75)	20 (7.41)	
65–74% (2 score)	43 (4.74)	33 (5.18)	10 (3.70)	
<65% (3 score)	28 (3.09)	14 (2.20)	14 (5.19)	
Sleep disturbance, *n* (%)				0.53
Not at all (0 score)	75 (8.27)	51 (8.01)	24 (8.89)	
1–9 (1 score)	669 (73.76)	473 (74.25)	196 (72.59)	
10–18 (2 score)	160 (17.64)	112 (17.58)	48 (17.78)	
19–28 (3 score)	3 (0.33)	1 (0.16)	2 (0.74)	
Hypnotic drug use, *n* (%)				0.246
Not during the past month (0 score)	887 (97.79)	619 (97.17)	268 (99.26)	
Less than once a week (1 score)	7 (0.77)	6 (0.94)	1 (0.37)	
Once or twice a week (2 score)	8 (0.88)	7 (1.10)	1 (0.37)	
Three or more times a week (3 score)	5 (0.55)	5 (0.78)	0 (0.00)	
Daytime disfunction, *n* (%)				**0.004**
Not at all (0 score)	322 (35.50)	234 (36.73)	88 (32.59)	
1–2 times (1 score)	255 (28.11)	188 (29.51)	67 (24.81)	
3–4 times (2 score)	232 (25.58)	161 (25.27)	71 (26.30)	
5–6 times (3 score)	98 (10.80)	54 (8.48)	44 (16.30)	
PSQI, good, *n* (%)	534 (58.88)	385 (60.44)	149 (55.19)	0.163

Data are mean (SD), median [interquartile range], or *n* (%). *p* value of continuous variables are calculated by Student’s t-test or Mann–Whitney U test, and categorical variables are calculated by *χ*^2^ test or Wilcoxon rank-sum test. NC, normal cognition; MCI, mild cognitive impairment; CHD, Coronary heart disease; BMI, body mass index; WHR, waist-hip ratio; SBP, systolic blood pressure; DBP, diastolic blood pressure; TMAO, trimethylamine N-oxide; ALT, alanine aminotransferase; GLU, fasting blood glucose; TCHOL, total cholesterol; HDL, high-density lipoprotein cholesterol; LDL, low-density lipoprotein cholesterol (LDL-C), PSQI, Pittsburgh sleep quality index.

### 3.2 Feature selection

**Figure 2:**
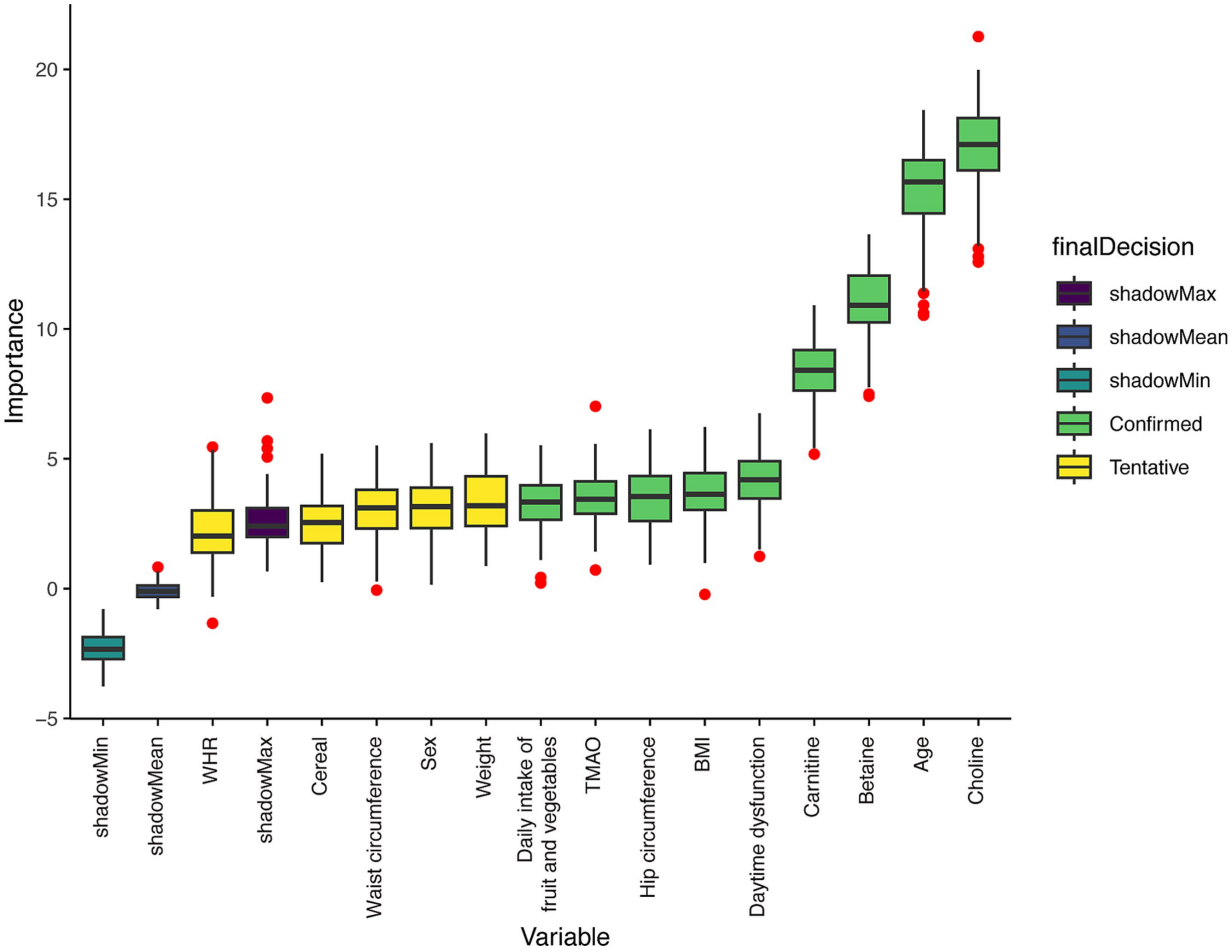
Importance of potential risk factors of MCI occurrence ranked by Boruta algorithm. The horizontal axis is the name of each variable, and the vertical axis is the Z value of each variable. The box plot shows the Z value of each variable during model calculation. The green boxes represent important variables, and the yellow boxes represent potentially important variables. WHR waist-hip ratio, TMAO trimethylamine N-oxide, BMI body mass index.

**Figure 3:**
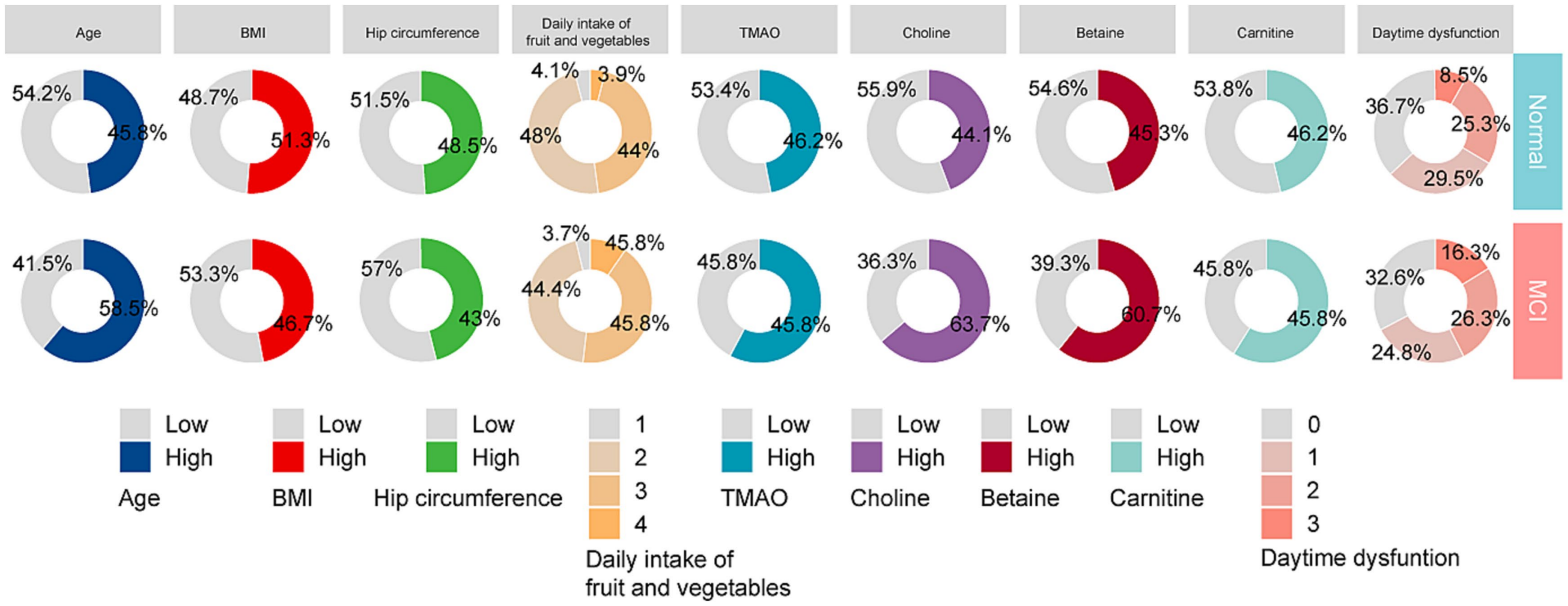
Comparison of nine predictors distribution among NC and MCI groups. Age, BMI, hip circumference, TMAO, choline, betaine, and carnitine were divided into low and high groups according to the median value. BMI body mass index, TMAO trimethylamine N-oxide.

In the report from Boruta algorithm, the variables including choline in the green area are identified as important factors, which have important roles in the model. Choline was one of the key factors in predicting MCI occurrence. The variables in the yellow area are suspected factors, which may be related to the adverse outcome to a certain extent, and the variables in the yellow area are unimportant factors (Figure 2). Ultimately, nine key predictors were brought into further analysis, including choline, age, betaine, carnitine, daytime dysfunction, BMI, hip circumference, TMAO, and daily intake of fruit and vegetables (Figure 2). The distributions of these predictors were then compared between the NC and MCI groups (Figure 3).

### 3.3 Development and validation of the MCI prediction model

**Figure 4:**
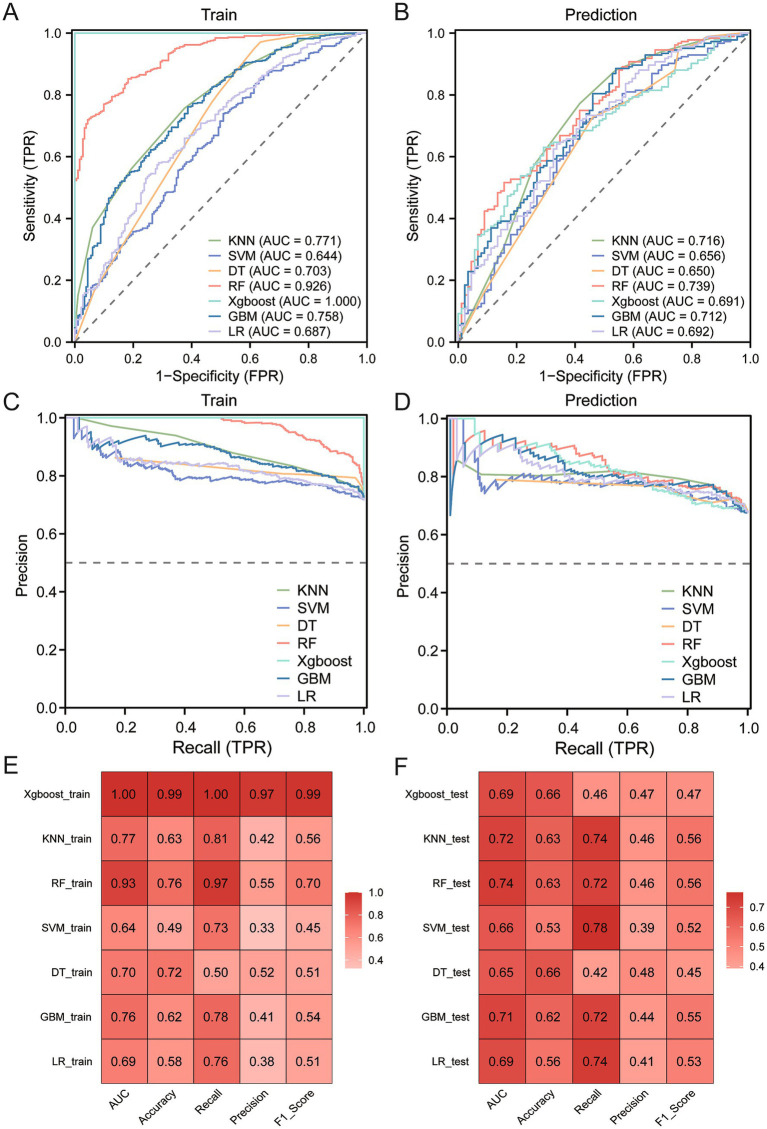
Development and validation of the MCI prediction models. ROC curves of diagnostic models developed by training cohort **(A)** and validation cohort **(B)**; PR curves of models developed by training cohort **(C)** and validation cohort **(D)**. Prediction performance of seven models by training cohort **(E)** and validation cohort **(F)**.

Herein, seven machine learning models were developed and evaluated through AUC. Table 2 presents a detailed evaluation of five ML models—XGBoost, GBM, RF, KNN, SVM, DT, and LR assessed on several performance metrics including AUC, accuracy, recall, precision, and F1 score. Considering the superiority of machine learning, the AUC values of all models exceeded 0.64, and the AUC values of RF reached 0.93 (95% CI: 0.906–0.945) and 0.74 (95% CI: 0.677–0.801) in the training cohort and the validation cohort, respectively (Figures 4A,B). However, given that the distribution of positive and negative events in the dataset was uneven, AUC alone was not sufficient to explain the performance of the model. Therefore, the PR curve was generated to make up for the inadequacy of the receiver operating characteristic (ROC) curve, thereby further evaluating the strengths and weaknesses of the model. It is evident seen that the average precision of the accuracy of the RF model was higher than that of other models (Figures 4C,D). Finally, the calibration curve was drawn to compare the discrimination of each model, with results showing that the RF model (training set: AUC = 0.93, accuracy = 0.76, recall = 0.97, precision = 0.55, F1 score = 0.70; testing set: AUC = 0.74, accuracy = 0.63, recall = 0.72, precision = 0.46, F1 score = 0.56) still maintained the best state (Figures 4E,F).

**Table 2:** The performance of predictive models on seven ML algorithms.

Models	Training cohort					Validation cohort				
	AUC	Accuracy	Recall	Precision	F1-score	AUC	Accuracy	Recall	Precision	F1-score
XGBoost	1.00	0.99	1.00	0.97	0.99	0.69	0.66	0.46	0.47	0.47
GBM	0.76	0.62	0.78	0.41	0.54	0.71	0.62	0.72	0.44	0.55
RF	0.93	0.76	0.97	0.55	0.70	0.74	0.63	0.72	0.46	0.56
KNN	0.77	0.63	0.81	0.42	0.56	0.72	0.63	0.74	0.46	0.56
SVM	0.64	0.49	0.73	0.33	0.45	0.66	0.53	0.78	0.39	0.52
DT	0.70	0.72	0.50	0.52	0.51	0.65	0.66	0.42	0.48	0.45
LR	0.69	0.58	0.76	0.38	0.51	0.69	0.56	0.74	0.41	0.53

XGBoost, Extreme Gradient Boosting; GBM, Gradient Boosting Machine; RF, Random Forest; KNN, k-Nearest Neighbors; SVM, Support Vector Machines; DT, Decision Tree; LR, Logistic Regression.

### 3.4 Importance of features interpreted by SHA*p* value

**Figure 5:**
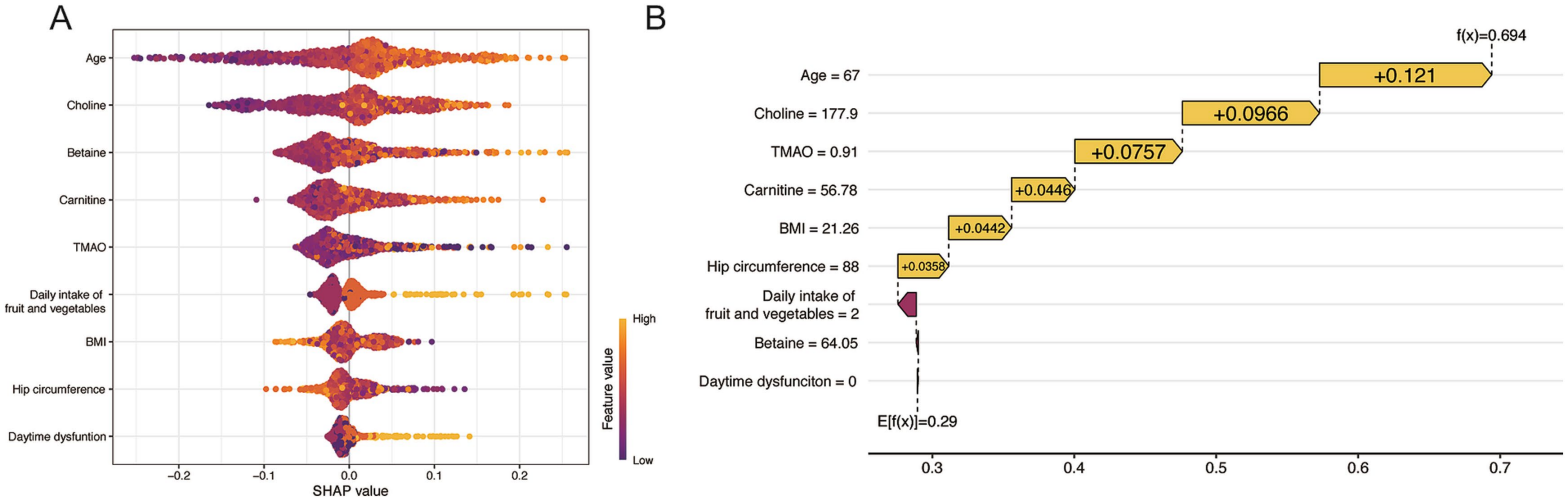
Explanation of the interpretability of the RF model (the best-performing model) for predicting MCI. **(A)** SHAP summary plot shows the top 9 risk predictors for prediction of MCI. **(B)** SHAP waterfall plot shows the prediction of a subject. The f(x) is the predicted value of each observation. TMAO trimethylamine N-oxide, BMI body mass index.

The SHAP plot (Figure 5A) offers a detailed exposition of the RF for MCI individuals, featuring the 9 most influential risk predictors. SHAP values indicate that age, choline, betaine, carnitine, TMAO, daily intake of fruit and vegetables, and daytime dysfunction were the positive contributors, indicating increasing the likelihood of cognitive impairment, while, BMI and hip circumference were the major negative contributors, indicating a decrease. A higher SHAP value for a given feature corresponds to a high level of importance in the predictive model. Notably, TMAO and its dietary precursors (choline, betaine, and carnitine) consistently exhibited strong positive SHAP effects, suggesting that gut microbiota–derived metabolites reflecting dietary protein and lipid intake play a biologically relevant role in distinguishing individuals at risk of MCI.

Figure 5B illustrates a specific case study, showing how the SHAP value for a particular individual (in this case, an adult aged 67 years) contribute to the RF’s prediction of MCI. Each feature has a predicative value, and the yellow ones indicating they push the prediction toward a higher risk of MCI. This individual was classified correctly as MCI with final prediction reaching 0.694, primarily due to older age, high expression of choline and TMAO, with smaller contributions from several other features, such as lower BMI and hip circumference. These visualizations provide users with detailed insights into how the model makes predictions, allowing them to pay attention to the vital risk factors.

## 4 Discussion

The predictive performance of the developed models was moderate but consistent with existing evidence for community-based early-stage MCI prediction models. The RF model achieved an AUC of 0.739 (95% CI: 0.677–0.801) and a recall (sensitivity) of 0.72 in the test set, indicating statistically significant discrimination above the random level (AUC = 0.5) and acceptable ability to identify individuals at risk. Such performance is expected for early MCI screening, where physiological and behavioral alterations are still subtle. In comparison, studies predicting MCI-to- AD conversion—a task with more distinct clinical phenotypes—typically report higher AUCs (0.75–0.85). Conversely, community-based MCI onset prediction remains challenging: a recent UK Biobank study (*N* = 126,785) integrating the Life’s Essential 8 (LE8) score with demographic factors achieved an AUC of 0.753 (Wang et al., 2024), comparable to our results despite its far larger sample size. These findings indicate that the current model performs within the expected range for early-stage MCI prediction and provides a feasible, interpretable framework for scalable screening in resource-limited settings.

This study leverages machine learning to integrate nine diverse predictors for forecasting cognitive decline, producing findings that align with existing literature. The model identified age, TMAO and its dietary precursors (choline, betaine, and carnitine), daily fruit and vegetable intake, BMI, hip circumference, and daytime dysfunction as key factors influencing cognitive function. These predictors are consistent with previous studies, supporting the robustness and relevance of the analytical framework (Tan et al., 2023; Zhang et al., 2025).

Consistent with prior findings, advanced age has been identified as a significant risk factor for cognitive impairment (Grueso and Viejo-Sobera, 2021). Accumulating evidence indicates that gut microbiota-derived metabolites such as TMAO play a critical role in cognitive health. Previous studies, including our own research, have consistently shown that elevated serum TMAO and its dietary precursors are associated with increased risk of mild cognitive impairment and cognitive decline (Long et al., 2024; Bai et al., 2024). These findings support the biological plausibility of TMAO-related metabolites as biomarkers reflecting gut–brain axis activity and neuroinflammatory processes.

A large proportion of the initial cohort lacked measurements of TMAO and its dietary precursors (choline, betaine, and carnitine). As shown in Supplementary Table S9, these metabolites had the highest missing rates (up to 59.96%), whereas all other variables had missing rates between 0.03 and 4.74%. To assess whether the exclusion of TMAO-related metabolites affected model performance, we conducted a comparative analysis using the same feature selection and modeling procedures but without TMAO and its precursors. As illustrated in Supplementary Figure S1, removing the TMAO-related inclusion criteria expanded the sample to N = 3,173. Feature selection based on the Boruta algorithm (Supplementary Table S7) was then applied to this dataset, and the corresponding model performance metrics are summarized in Supplementary Table S8. Across all algorithms, the discriminative performance decreased notably when TMAO-related metabolites were excluded—for example, the AUC of the RF model in the test set declined from 0.739 to 0.656. These results indicate that while other low-missing-rate variables are important, they cannot replace the unique biological and predictive contribution provided by TMAO and its precursors. Furthermore, TMAO and its precursors were consistently identified as key predictors by three independent feature-selection algorithms (Boruta, LASSO, and SVM-RFE; Supplementary Table S2), confirming the robustness and non-random nature of their inclusion and underscoring their biological and statistical relevance to MCI risk.

To determine whether the higher missing rate of TMAO-related data introduced selection bias, we compared demographic, lifestyle, and clinical characteristics between participants with and without TMAO measurements. As shown in Supplementary Table S10, there were no statistically significant differences across key baseline variables between the two groups, suggesting that the subset of participants with biochemical data was representative of the overall cohort. This finding supports the assumption that the observed associations between TMAO-related metabolites and MCI risk are unlikely to be confounded by sampling bias. Therefore, although the inclusion of TMAO reduced the analytic sample size, it preserved biological validity, model interpretability, and generalizability within the studied population.

From a biological perspective, TMAO and its precursors are metabolites of dietary protein and lipid intake that reflect gut–brain axis activity. Previous studies have demonstrated that elevated TMAO levels are associated with neuroinflammation, oxidative stress, and increased risk of MCI (Zhou et al., 2023; Xu et al., 2022). Taken together, the consistent predictive contribution and biological plausibility of TMAO-related metabolites suggest that incorporating them into the model not only improves interpretability but also provides novel mechanistic insights into the nutritional and metabolic pathways underlying early cognitive decline. In future work, expanding biochemical and lifestyle data collection in larger and more diverse samples will help further improve model performance, robustness, and generalizability.

Additionaly, in our study, higher daily consumption of fruit and vegetables was a significant predictor for cognitive decline. This finding is consistent with previous research showing that high fruit intake is linked to elevated C-reactive protein levels, which may increase the risk of cognitive impairment in patients undergoing hemodialysis (Zhuang et al., 2023). However, this result contrasts with the more commonly reported protective effects of fruit and vegetable consumption on cognitive health, suggesting that the relationship may vary depending on individual health conditions, dietary patterns, or population characteristics.

BMI and hip circumference also emerged as significant predictors of cognitive impairment (Li et al., 2023). We observed that underweight individuals were more vulnerable to MCI, in line with study indicating that low BMI (<23 kg/m^2^) was associated with a higher risk of cognitive decline such as Alzheimer’s disease (Yuan et al., 2022). Conversely, other studies, such as that by Aschwanden et al., have reported that higher BMI predicts worse cognitive outcomes (Lee et al., 2023). These conflicting findings highlight the complexity of the association between body composition and cognitive health, which may be influenced by age, metabolic status, fat distribution, and underlying health conditions. Further investigation is needed to clarify the role of BMI in cognitive aging across different populations.

Sleep quality has previously been identified as a significant predictor of MCI, with daytime dysfunction—a specific component of sleep quality—playing a particularly prominent role (Hu et al., 2025). Consistent with these findings, our study further demonstrated that greater daytime dysfunction was significantly associated with an increased risk of MCI. This association may be explained by mechanisms such as the accumulation of neurotoxic proteins (e.g., beta-amyloid) and disruptions in cognitive processes like memory consolidation and synaptic plasticity (Blackman et al., 2021; Yoon et al., 2022; Huang et al., 2021). The inclusion of daytime dysfunction in our predictive model underscores its importance as a modifiable risk factor and highlights the multifactorial nature of MCI.

Although most SHAP values were concentrated near zero, this distribution does not imply a lack of predictive contribution. In the SHAP framework, correlated predictors tend to share their marginal effects, leading to smaller absolute values while maintaining consistent directional influence. This pattern is expected in early-stage MCI prediction, where physiological and behavioral differences are subtle and interrelated. The consistently positive contributions of age, TMAO and its precursors, and daytime dysfunction, together with the negative influence of BMI and hip circumference, further confirm the directional stability and interpretability of the model.

Collectively, our findings highlight the complementary role of metabolic, behavioral, and physical indicators in explaining early cognitive changes. By integrating these multidimensional data using interpretable ML algorithms, the study advances a feasible framework for scalable MCI risk stratification in real-world settings. Traditional screening tools for cognitive impairment—such as the MoCA or MMSE—remain limited by their reliance on subjective reporting, clinical expertise, and time-consuming administration. In contrast, our approach enables rapid, objective assessment by combining routinely collected physiological measures with biochemical markers such as TMAO, offering a cost-effective and data-driven alternative for community-level screening.

Importantly, the integration of TMAO-related biomarkers into public health platforms has practical implications for early intervention. For example, China’s National Basic Public Health Service Program provides free annual health checkups for older adults aged 65 years and above, offering a feasible infrastructure to incorporate metabolic biomarkers into population-level screening. Embedding TMAO and its dietary precursors into these programs could facilitate early identification of individuals at elevated cognitive risk and inform targeted preventive strategies.

This study has several limitations that should be acknowledged. First, the sample size was relatively modest and drawn from a single rural county, which may limit the statistical power and generalizability of the findings. Second, the cross-sectional design precludes inference about causality between metabolic, behavioral, and cognitive variables. Third, some predictors—particularly daytime dysfunction—were self-reported and thus subject to recall or reporting bias. Fourth, missing data for TMAO and related metabolites reduced the analyzable sample, although the inclusion of a core model without these variables yielded consistent results. Fifth, although multiple imputation and nested cross-validation were applied to enhance robustness, residual uncertainty due to imputation and internal validation remains possible. Finally, the models were internally validated only; external or temporal validation in larger, multi-center cohorts is warranted to confirm reproducibility and generalizability.

Despite these limitations, this study also has notable strengths. It integrates objective biochemical biomarkers with behavioral and physical indicators using explainable machine-learning techniques, providing a multidimensional view of early-stage cognitive decline. The work represents one of the few community-based studies to explore MCI onset prediction rather than MCI-to-AD conversion, demonstrating methodological feasibility for scalable, low-cost screening in resource-limited settings. Together, these strengths lay the groundwork for future longitudinal and multi-center research aimed at developing dynamic, personalized risk-prediction tools for cognitive impairment.

In conclusion, this study demonstrates the potential of ML models not only to predict the risk of MCI but also to identify biologically meaningful predictors by integrating age, serum biomarkers (TMAO and its precursors), physical examination, dietary habits, and daytime dysfunction. TMAO-related metabolites consistently emerged as important contributors, suggesting that gut microbiota–derived nutritional biomarkers can provide mechanistic insight into early cognitive decline. These results highlight the value of combining biochemical and lifestyle factors to enhance the understanding and early identification of cognitive impairment. Future research should expand biochemical and lifestyle data collection across larger and more diverse populations, integrate causal and longitudinal analyses, and further explore the translational potential of TMAO-related biomarkers for early prevention and intervention in cognitive aging.

## Data Availability

The raw data supporting the conclusions of this article will be made available by the authors, without undue reservation.
