# Supplementary material for: Machine learning-based early screening of mild cognitive impairment using nutrition-related biomarkers and functional indicators
Source: Front Aging Neurosci. 2025 Dec 5;17:1641690. doi: 10.3389/fnagi.2025.1641690 (PMC12714913; doi:10.3389/fnagi.2025.1641690)
Supplement: Supplementary file 1 [file Data_Sheet_1.pdf]

Supplementary Materials

Figure S1. Flow diagram for the inclusion and exclusion of participants (Remove TMAO variable as a screening criterion, N = 3171)

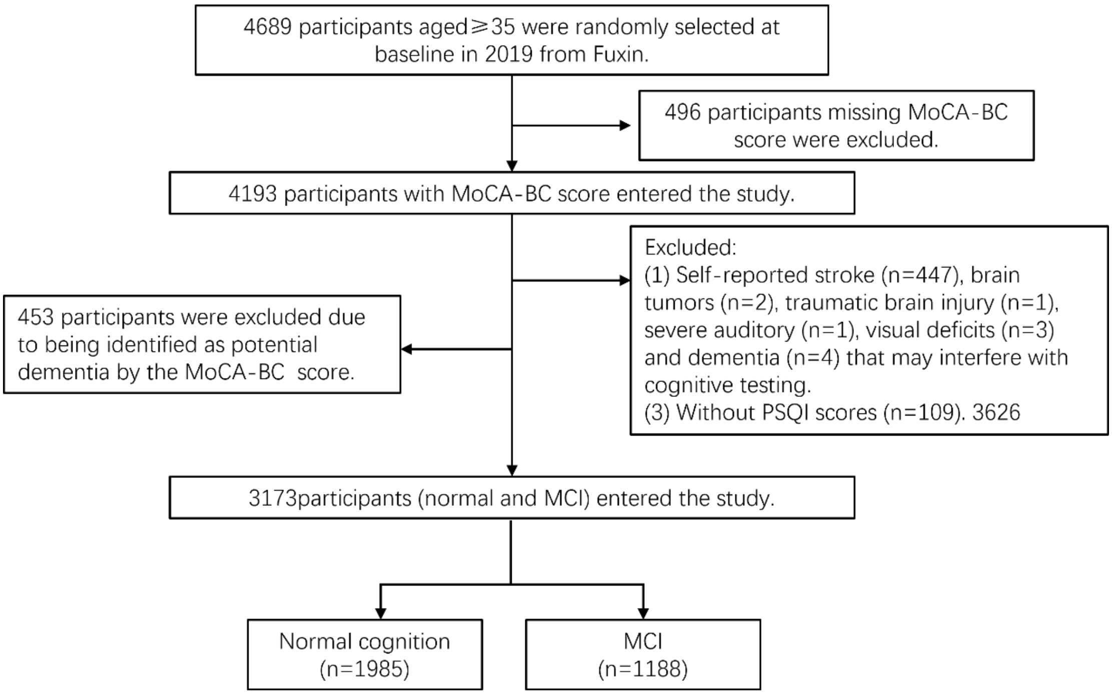

Table S1. Summary of missingness patterns and descriptive statistics of variables before and after imputation (N = 1452, including normal, MCI and dementia)

variables	n	Missing_Count	Missing_Rate	mean	sd	median	trimmed	mad	min	max	range	skew	kurtosis	se	Dataset
MoCA	1452	0	0.00	0.53	0.68	0.00	0.41	0.00	0.00	2.00	2.00	0.92	-0.38	0.02	Original
Age	1452	0	0.00	58.89	9.36	60.00	59.11	10.38	35.00	83.00	48.00	-0.18	-0.63	0.25	Original
Sex	1452	0	0.00	1.68	0.47	2.00	1.73	0.00	1.00	2.00	1.00	-0.78	-1.39	0.01	Original
Family members	1436	16	1.10	3.05	1.38	3.00	2.94	1.48	1.00	9.00	8.00	0.71	-0.41	0.04	Original
Education	1452	0	0.00	0.76	0.71	1.00	0.70	1.48	0.00	2.00	2.00	0.38	-0.97	0.02	Original
Marriage	1450	2	0.14	0.11	0.36	0.00	0.00	0.00	0.00	2.00	2.00	3.26	10.61	0.01	Original
Ethnic	1451	1	0.07	0.39	0.56	0.00	0.32	0.00	0.00	2.00	2.00	1.07	0.13	0.01	Original
Income	1448	4	0.28	0.29	0.45	0.00	0.23	0.00	0.00	1.00	1.00	0.94	-1.11	0.01	Original
Diabetes	1452	0	0.00	0.08	0.26	0.00	0.00	0.00	0.00	1.00	1.00	3.20	8.27	0.01	Original
Hypertension	1452	0	0.00	0.28	0.45	0.00	0.23	0.00	0.00	1.00	1.00	0.98	-1.05	0.01	Original
CHD	1452	0	0.00	0.14	0.35	0.00	0.06	0.00	0.00	1.00	1.00	2.02	2.08	0.01	Original
High	1451	1	0.07	160.25	7.53	160.00	160.04	7.41	138.00	183.00	45.00	0.25	-0.20	0.20	Original
Weight	1451	1	0.07	63.51	11.06	63.00	63.10	11.12	34.00	108.00	74.00	0.39	0.28	0.29	Original
BMI	1451	1	0.07	24.68	3.67	24.53	24.56	3.69	14.53	39.84	25.32	0.38	0.32	0.10	Original
Waist circumference	1451	1	0.07	84.03	9.93	84.00	84.03	10.38	50.00	122.00	72.00	0.03	0.11	0.26	Original
Hip circumference	1451	1	0.07	94.86	8.01	95.00	94.78	7.41	41.00	188.00	147.00	0.68	15.39	0.21	Original
WHR	1451	1	0.07	0.89	0.08	0.89	0.89	0.06	0.41	1.88	1.47	2.74	34.98	0.00	Original
SBP	1447	5	0.34	133.42	20.50	132.00	132.12	20.26	87.67	254.67	167.00	0.70	0.94	0.54	Original
DBP	1447	5	0.34	80.06	10.68	79.67	79.79	10.87	49.67	119.67	70.00	0.26	-0.03	0.28	Original

Heart rate	1446	6	0.41	75.50	11.25	74.00	74.90	10.38	46.67	129.00	82.33	0.64	0.87	0.30	Original
Daily salt intake	1332	120	8.26	20.45	12.40	16.67	18.83	8.24	0.56	125.00	124.44	2.54	11.96	0.34	Original
Daily oil intake	1395	57	3.93	148.16	68.32	166.67	141.92	61.78	5.54	500.00	494.46	1.14	2.89	1.83	Original
Weight management	1334	118	8.13	0.07	0.25	0.00	0.00	0.00	0.00	1.00	1.00	3.52	10.38	0.01	Original
Current smoker	1450	2	0.14	1.66	0.47	2.00	1.70	0.00	1.00	2.00	1.00	-0.69	-1.52	0.01	Original
Current drinker	1449	3	0.21	0.21	0.40	0.00	0.13	0.00	0.00	1.00	1.00	1.45	0.10	0.01	Original
Home passive smoker	1436	16	1.10	1.64	0.48	2.00	1.68	0.00	1.00	2.00	1.00	-0.59	-1.65	0.01	Original
Tea	1449	3	0.21	1.46	0.50	1.00	1.46	0.00	1.00	2.00	1.00	0.14	-1.98	0.01	Original
Daily intake of fruit and vegetables	1442	10	0.69	2.48	0.68	2.00	2.46	1.48	1.00	4.00	3.00	0.20	-0.21	0.02	Original
Weekly intake of fish	1437	15	1.03	1.40	0.57	1.00	1.32	0.00	1.00	4.00	3.00	1.16	0.58	0.02	Original
Weekly intake of sugar	1428	24	1.65	1.36	0.55	1.00	1.28	0.00	1.00	4.00	3.00	1.32	1.18	0.01	Original
Weekly intake of beverage	1391	61	4.20	1.05	0.24	1.00	1.00	0.00	1.00	3.00	2.00	4.87	25.22	0.01	Original
Cereal	1442	10	0.69	2.75	1.11	3.00	2.81	1.48	1.00	4.00	3.00	-0.32	-1.26	0.03	Original
Physical labour level	1442	10	0.69	1.76	0.51	2.00	1.78	0.00	1.00	3.00	2.00	-0.31	-0.19	0.01	Original
Moderate intensity exercise	1416	36	2.48	0.41	0.77	0.00	0.26	0.00	0.00	2.00	2.00	1.46	0.29	0.02	Original
TMAO	1452	0	0.00	6.14	9.49	3.96	4.69	3.34	0.00	252.92	252.92	13.57	319.03	0.25	Original
Choline	1452	0	0.00	201.06	126.65	179.14	187.06	84.58	6.08	2489.02	2482.93	5.10	74.74	3.32	Original
Betaine	1452	0	0.00	115.40	68.03	100.53	107.53	47.35	2.74	540.66	537.92	1.78	5.53	1.79	Original
Carnitine	1452	0	0.00	56.42	26.55	50.28	53.77	18.95	3.66	207.28	203.62	1.16	2.29	0.70	Original
Subjective sleep quality	1452	0	0.00	0.91	0.82	1.00	0.84	1.48	0.00	3.00	3.00	0.63	-0.19	0.02	Original
Sleep onset latency	1452	0	0.00	0.94	1.03	1.00	0.80	1.48	0.00	3.00	3.00	0.77	-0.65	0.03	Original
Sleep duration	1452	0	0.00	1.23	0.97	1.00	1.16	1.48	0.00	3.00	3.00	0.33	-0.87	0.03	Original

Sleep efficiency	1452	0	0.00	0.29	0.73	0.00	0.08	0.00	0.00	3.00	3.00	2.61	5.89	0.02	Original
Sleep disturbance	1452	0	0.00	1.11	0.52	1.00	1.11	0.00	0.00	3.00	3.00	0.32	1.00	0.01	Original
Hypnotic drug use	1452	0	0.00	0.05	0.33	0.00	0.00	0.00	0.00	3.00	3.00	7.38	55.77	0.01	Original
Daytime disfunction	1452	0	0.00	1.12	1.02	1.00	1.02	1.48	0.00	3.00	3.00	0.42	-1.02	0.03	Original
PSQI	1452	0	0.00	0.41	0.49	0.00	0.39	0.00	0.00	1.00	1.00	0.35	-1.88	0.01	Original
ALT	1452	0	0.00	0.04	0.21	0.00	0.00	0.00	0.00	1.00	1.00	4.40	17.36	0.01	Original
GLU	1452	0	0.00	0.24	0.43	0.00	0.17	0.00	0.00	1.00	1.00	1.23	-0.48	0.01	Original
TCHOL	1452	0	0.00	0.48	0.50	0.00	0.47	0.00	0.00	1.00	1.00	0.09	-1.99	0.01	Original
HDL	1452	0	0.00	0.28	0.45	0.00	0.23	0.00	0.00	1.00	1.00	0.97	-1.06	0.01	Original
LDL	1452	0	0.00	0.45	0.50	0.00	0.44	0.00	0.00	1.00	1.00	0.19	-1.96	0.01	Original
Creatinine	1452	0	0.00	0.03	0.17	0.00	0.00	0.00	0.00	1.00	1.00	5.62	29.56	0.00	Original
Uric acid	1452	0	0.00	0.06	0.24	0.00	0.00	0.00	0.00	1.00	1.00	3.63	11.18	0.01	Original
MoCA	1452	0	0.00	0.53	0.68	0.00	0.41	0.00	0.00	2.00	2.00	0.92	-0.38	0.02	Imputed
Age	1452	0	0.00	58.89	9.36	60.00	59.11	10.38	35.00	83.00	48.00	-0.18	-0.63	0.25	Imputed
Sex	1452	0	0.00	1.68	0.47	2.00	1.73	0.00	1.00	2.00	1.00	-0.78	-1.39	0.01	Imputed
Family members	1452	0	0.00	3.05	1.38	3.00	2.94	1.48	1.00	9.00	8.00	0.71	-0.41	0.04	Imputed
Education	1452	0	0.00	0.76	0.71	1.00	0.70	1.48	0.00	2.00	2.00	0.38	-0.97	0.02	Imputed
Marriage	1452	0	0.00	0.11	0.36	0.00	0.00	0.00	0.00	2.00	2.00	3.26	10.63	0.01	Imputed
Ethnic	1452	0	0.00	0.39	0.56	0.00	0.32	0.00	0.00	2.00	2.00	1.06	0.13	0.01	Imputed
Income	1452	0	0.00	0.29	0.45	0.00	0.23	0.00	0.00	1.00	1.00	0.94	-1.11	0.01	Imputed
Diabetes	1452	0	0.00	0.08	0.26	0.00	0.00	0.00	0.00	1.00	1.00	3.20	8.27	0.01	Imputed
Hypertension	1452	0	0.00	0.28	0.45	0.00	0.23	0.00	0.00	1.00	1.00	0.98	-1.05	0.01	Imputed
CHD	1452	0	0.00	0.14	0.35	0.00	0.06	0.00	0.00	1.00	1.00	2.02	2.08	0.01	Imputed
High	1452	0	0.00	160.25	7.53	160.00	160.04	7.41	138.00	183.00	45.00	0.25	-0.19	0.20	Imputed
Weight	1452	0	0.00	63.51	11.06	63.00	63.10	11.12	34.00	108.00	74.00	0.39	0.28	0.29	Imputed

BMI	1452	0	0.00	24.68	3.67	24.54	24.56	3.70	14.53	39.84	25.32	0.38	0.33	0.10	Imputed
Waist circumference	1452	0	0.00	84.03	9.92	84.00	84.03	10.38	50.00	122.00	72.00	0.03	0.12	0.26	Imputed
Hip circumference	1452	0	0.00	94.86	8.01	95.00	94.78	7.41	41.00	188.00	147.00	0.68	15.40	0.21	Imputed
WHR	1452	0	0.00	0.89	0.08	0.89	0.89	0.06	0.41	1.88	1.47	2.74	34.99	0.00	Imputed
SBP	1452	0	0.00	133.41	20.48	132.00	132.11	20.26	87.67	254.67	167.00	0.70	0.95	0.54	Imputed
DBP	1452	0	0.00	80.05	10.67	79.67	79.78	11.12	49.67	119.67	70.00	0.26	-0.03	0.28	Imputed
Heart rate	1452	0	0.00	75.47	11.26	74.00	74.88	10.38	46.67	129.00	82.33	0.64	0.87	0.30	Imputed
Daily salt intake	1452	0	0.00	20.32	12.24	16.67	18.72	8.24	0.56	125.00	124.44	2.50	11.74	0.32	Imputed
Daily oil intake	1452	0	0.00	147.88	67.90	166.67	141.55	61.78	5.54	500.00	494.46	1.15	2.92	1.78	Imputed
Weight management	1452	0	0.00	0.06	0.24	0.00	0.00	0.00	0.00	1.00	1.00	3.56	10.66	0.01	Imputed
Current smoker	1452	0	0.00	1.66	0.47	2.00	1.70	0.00	1.00	2.00	1.00	-0.69	-1.52	0.01	Imputed
Current drinker	1452	0	0.00	0.21	0.41	0.00	0.13	0.00	0.00	1.00	1.00	1.45	0.10	0.01	Imputed
Home passive smoker	1452	0	0.00	1.64	0.48	2.00	1.68	0.00	1.00	2.00	1.00	-0.59	-1.65	0.01	Imputed
Tea	1452	0	0.00	1.46	0.50	1.00	1.45	0.00	1.00	2.00	1.00	0.15	-1.98	0.01	Imputed
Daily intake of fruit and vegetables	1452	0	0.00	2.48	0.68	2.00	2.46	1.48	1.00	4.00	3.00	0.20	-0.21	0.02	Imputed
Weekly intake of fish	1452	0	0.00	1.40	0.58	1.00	1.32	0.00	1.00	4.00	3.00	1.15	0.56	0.02	Imputed
Weekly intake of sugar	1452	0	0.00	1.36	0.55	1.00	1.28	0.00	1.00	4.00	3.00	1.32	1.19	0.01	Imputed
Weekly intake of beverage	1452	0	0.00	1.06	0.25	1.00	1.00	0.00	1.00	3.00	2.00	4.70	23.47	0.01	Imputed
Cereal	1452	0	0.00	2.75	1.11	3.00	2.81	1.48	1.00	4.00	3.00	-0.33	-1.26	0.03	Imputed
Physical labour level	1452	0	0.00	1.76	0.51	2.00	1.78	0.00	1.00	3.00	2.00	-0.30	-0.21	0.01	Imputed
Moderate intensity exercise	1452	0	0.00	0.41	0.77	0.00	0.26	0.00	0.00	2.00	2.00	1.46	0.27	0.02	Imputed
TMAO	1452	0	0.00	6.14	9.49	3.96	4.69	3.34	0.00	252.92	252.92	13.57	319.03	0.25	Imputed

Choline	1452	0	0.00	201.06	126.65	179.14	187.06	84.58	6.08	2489.02	2482.93	5.10	74.74	3.32	Imputed
Betaine	1452	0	0.00	115.40	68.03	100.53	107.53	47.35	2.74	540.66	537.92	1.78	5.53	1.79	Imputed
Carnitine	1452	0	0.00	56.42	26.55	50.28	53.77	18.95	3.66	207.28	203.62	1.16	2.29	0.70	Imputed
Subjective sleep quality	1452	0	0.00	0.91	0.82	1.00	0.84	1.48	0.00	3.00	3.00	0.63	-0.19	0.02	Imputed
Sleep onset latency	1452	0	0.00	0.94	1.03	1.00	0.80	1.48	0.00	3.00	3.00	0.77	-0.65	0.03	Imputed
Sleep duration	1452	0	0.00	1.23	0.97	1.00	1.16	1.48	0.00	3.00	3.00	0.33	-0.87	0.03	Imputed
Sleep efficiency	1452	0	0.00	0.29	0.73	0.00	0.08	0.00	0.00	3.00	3.00	2.61	5.89	0.02	Imputed
Sleep disturbance	1452	0	0.00	1.11	0.52	1.00	1.11	0.00	0.00	3.00	3.00	0.32	1.00	0.01	Imputed
Hypnotic drug use	1452	0	0.00	0.05	0.33	0.00	0.00	0.00	0.00	3.00	3.00	7.38	55.77	0.01	Imputed
Daytime disfunction	1452	0	0.00	1.12	1.02	1.00	1.02	1.48	0.00	3.00	3.00	0.42	-1.02	0.03	Imputed
PSQI	1452	0	0.00	0.41	0.49	0.00	0.39	0.00	0.00	1.00	1.00	0.35	-1.88	0.01	Imputed
ALT	1452	0	0.00	0.04	0.21	0.00	0.00	0.00	0.00	1.00	1.00	4.40	17.36	0.01	Imputed
GLU	1452	0	0.00	0.24	0.43	0.00	0.17	0.00	0.00	1.00	1.00	1.23	-0.48	0.01	Imputed
TCHOL	1452	0	0.00	0.48	0.50	0.00	0.47	0.00	0.00	1.00	1.00	0.09	-1.99	0.01	Imputed
HDL	1452	0	0.00	0.28	0.45	0.00	0.23	0.00	0.00	1.00	1.00	0.97	-1.06	0.01	Imputed
LDL	1452	0	0.00	0.45	0.50	0.00	0.44	0.00	0.00	1.00	1.00	0.19	-1.96	0.01	Imputed
Creatinine	1452	0	0.00	0.03	0.17	0.00	0.00	0.00	0.00	1.00	1.00	5.62	29.56	0.00	Imputed
Uric acid	1452	0	0.00	0.06	0.24	0.00	0.00	0.00	0.00	1.00	1.00	3.63	11.18	0.01	Imputed

MoCA Montreal cognitive assessment, *CHD* Coronary heart disease, *BMI* body mass index, *WHR* waist-hip ratio, *SBP* systolic blood pressure, *DBP* diastolic blood pressure, *TMAO* trimethylamine N-oxide, *ALT* alanine aminotransferase, *GLU* fasting blood glucose, *TCHOL* total cholesterol, *HDL* high-density lipoprotein cholesterol, *LDL* low-density lipoprotein cholesterol (LDL-C), *PSQI* Pittsburgh sleep quality index.

Table S2. Feature Selection Results Using Three Different Methods (N = 907)

Model	Selected Feature	Model	Selected Feature
Boruta	Choline		Tea
	Age		CHD
	Betaine		PSQI
	Carnitine		Home passive smoker
	Daytime disfunction		LDL
	BMI		HDL
	Hip		GLU
	TMAO		TCHOL
			Income
	Daily intake of fruit and vegetables	No Feature Selection	Age
LASSO	Age		Sex
	Sex		Family members
	Hip circumference		Education
	DBP		Marriage
	Heart rate		Ethnicity
	Daily salt intake		Income
	Daily oil intake		Diabetes
	Tea		Hypertension
	Daily intake of fruit and vegetables		CHD
	Cereal		High
	Choline		Weight
	Betaine		BMI
	Carnitine		Waist circumference
	ALT		Hip circumference
	Subjective sleep quality		WHR
	Sleep onset latency		SBP
	Hypnotic drug use		DBP
	Daytime disfunction		Heart rate
SVM-RFE	Choline		Daily salt intake
	Age		Daily oil intake
	Betaine		Weight management
	TMAO		Current smoker
	Carnitine		Current drinker
	Heart rate		Home passive smoker
	DBP		Tea
	WHR		Daily intake of fruit and vegetables
	SBP		Weekly intake of fish
	High		Weekly intake of sugar

BMI	Weekly intake of beverage
Weight	Weekly intake of Cereal
Hip circumference	Physical labour level
Waist circumference	Moderate intensity exercise
Daily salt intake	TMAO
Daily salt intake	Choline
Daytime disfunction	Betaine
Cereal	Carnitine
Family members	ALT
Daily intake of fruit and vegetables	GLU
Sleep onset latency	TCHOL
Sleep duration	HDL
Education	LDL
Subjective sleep quality	Creatinine
Weekly intake of sugar	Uric acid
Sleep efficiency	Subjective sleep quality
Sex	Sleep onset latency
Weekly intake of fish	Sleep duration
Sleep disturbance	Sleep efficiency
Ethnicity	Sleep disturbance
Moderate intensity exercise	Hypnotic drug use
Current smoker	Daytime disfunction
Hypertension	PSQI
Physical labour level	

CHD Coronary heart disease, *BMI* body mass index, *WHR* waist-hip ratio, *SBP* systolic blood pressure, *DBP* diastolic blood pressure, *TMAO* trimethylamine N-oxide, *ALT* alanine aminotransferase, *GLU* fasting blood glucose, *TCHOL* total cholesterol, *HDL* high-density lipoprotein cholesterol, *LDL* low-density lipoprotein cholesterol (LDL-C), *PSQI* Pittsburgh sleep quality index.

Table S3. Hyperparameters and Fitting Results with Confidence Intervals for Each Model with Boruta Feature Selection (N = 907)

Model	Hyperparameter		Train_AUC	Test AUC
SVM	cost	2.319097	0.644	0.656
	kernel	polynomial	(0.596, 0.692)	(0.585, 0.726)
	gamma	2.290557		
	type	C-classification		
	degree	1		
DT	xval	0	0.703	0.650
	cp	0.01103474	(0.657, 0.749)	(0.582, 0.717)
	minsplit	1		
	maxdepth	9		
	minbucket	6		
RF	num.threads	1	0.926	0.739
	num.trees	1274	(0.906, 0.945)	(0.677, 0.801)
	mtry	5		
	min.node.size	5		
	max.depth	5		
KNN	k	15	0.716	0.514
	distance	1.5907	(0.732, 0.810)	(0.648, 0.785)
	kernel	rectangular		
XGBoost	nrounds	800	1.000	0.691
	nthread	1	(1.000, 1.000)	(0.627, 0.755)
	verbose	0		
	eta	1		
	gamma	1		
	max_depth	5		
	subsample	0.9891309		
	eval_metric	mlogloss		
GBM	n.cores	1	0.712	0.667
	n.trees	1289	(0.716, 0.799)	(0.646, 0.778)
	n.minobsinnode	1		
	interaction.depth	12		
LR	-	-	0.687	0.692
			(0.641, 0.733)	(0.624, 0.759)

Table S4. Hyperparameters and Fitting Results with Confidence Intervals for Each Model with LASSO Feature Selection (N = 907)

Model	Hyperparameter		Train_AUC	Test AUC
SVM	cost	1.120653	0.703	0.708
	kernel	polynomial	(0.657, 0.748)	(0.642, 0.773)
	gamma	6.622656		
	type	C-classification		
	degree	1		
DT	xval	0	0.727	0.627
	cp	0.002448098	(0.684, 0.771)	(0.556, 0.699)
	minsplit	8		
	maxdepth	4		
	minbucket	6		
RF	num.threads	1	0.953	0.724
	num.trees	756	(0.937, 0.970)	(0.688, 0.785)
	mtry	1		
	min.node.size	1		
	max.depth	6		
KNN	k	15	0.777	0.665
	distance	1.7405	(0.738, 0.817)	(0.595, 0.735)
	kernel	rectangular		
XGBoost	nrounds	800	1.000	0.673
	nthread	1	(1.000, 1.000)	(0.604, 0.743)
	verbose	0		
	eta	1		
	gamma	2		
	max_depth	5		
	subsample	0.8088819		
	eval_metric	merror		
GBM	n.cores	1	0.767	0.746
	n.trees	501	(0.726, 0.808)	(0.684, 0.808)
	n.minobsinnode	5		
	interaction.depth	1		
LR	-	-	0.729	0.695
			(0.687, 0.771)	(0.627, 0.763)

Table S5. Hyperparameters and Fitting Results with Confidence Intervals for Each Model with SVM-RFE Feature Selection (N = 907)

Model	Hyperparameter		Train_AUC	Test AUC
SVM	cost	0.2038825	0.721	0.646
	kernel	polynomial	(0.675, 0.766)	(0.576, 0.716)
	gamma	1.410498		
	type	C-classification		
	degree	1		
DT	xval	0	0.806	0.620
	cp	0.01097464	(0.764, 0.848)	(0.547, 0.693)
	minsplit	4		
	maxdepth	7		
	minbucket	6		
RF	num.threads	1	1.000	0.713
	num.trees	577	(1.000, 1.000)	(0.645, 0.780)
	mtry	3		
	min.node.size	3		
	max.depth	10		
KNN	k	15	0.727	0.608
	distance	1.5907	(0.687, 0.767)	(0.536, 0.680)
	kernel	rectangular		
XGBoost	nrounds	800	1.000	0.683
	nthread	1	(1.000, 1.000)	(0.614, 0.752)
	verbose	0		
	eta	1		
	gamma	5		
	max_depth	1		
	subsample	0.9921252		
	eval_metric	mlogloss		
GBM	n.cores	1	0.785	0.717
	n.trees	744	(0.745, 0.824)	(0.652, 0.782)
	n.minobsinnode	3		
	interaction.depth	12		
LR	-	-	0.738	0.650
			(0.695, 0.780)	(0.580, 0.721)

Table S6. Hyperparameters and Fitting Results with Confidence Intervals for Each Model without Feature Selection (N = 907)

Model	Hyperparameter		Train_AUC	Test AUC
SVM	cost	0.1262177	0.727	0.646
	kernel	polynomial	(0.682, 0.772)	(0.576, 0.716)
	gamma	0.6693204		
	type	C-classification		
	degree	1		
DT	xval	0	0.809	0.62
	cp	0.01097464	(0.767, 0.851)	(0.551, 0.697)
	minsplit	4		
	maxdepth	7		
	minbucket	6		
RF	num.threads	1	0.998	0.717
	num.trees	542	(0.995, 1.000)	(0.651, 0.783)
	mtry	5		
	min.node.size	5		
	max.depth	7		
KNN	k	15	0.986	0.575
	distance	2.738822	(0.979, 0.993)	(0.502, 0.648)
	kernel	gaussian		
XGBoost	nrounds	800	1.000	0.716
	nthread	1	(1.000, 1.000)	(0.653, 0.778)
	verbose	0		
	eta	1		
	gamma	1		
	max_depth	5		
	subsample	0.99535		
	eval_metric	mlogloss		
GBM	n.cores	1	0.750	0.717
	n.trees	692	(0.745, 0.824)	(0.652, 0.782)
	n.minobsinnode	2		
	interaction.depth	13		
LR	-	-	0.749	0.640
			(0.708, 0.790)	(0.569, 0.710)

Table S7. Boruta Feature Selection Screening Results

Selected Features
Without TMAO (3173 participants)
Age
Sex
Education
High
Weight
BMI
Waist circumference
Hip circumference
WHR
SBP
Heart
Current smoker
Daily intake of fruit and vegetables
Moderate intensity exercise
Sleep onset latency
Daytime disfunction
With TMAO (907 participants)
Choline
Age
Betaine
Carnitine
Daytime disfunction
BMI
Hip circumference
TMAO
Daily intake of fruit and vegetables

BMI body mass index, *WHR* waist-hip ratio, *SBP* systolic blood pressure, *TMAO* trimethylamine N-oxide.

Table S8. Hyperparameters and Fitting Results with Confidence Intervals for Each Model With Boruta Feature Selection (Remove TMAO variable as a screening criterion, N=3173)

Model	Hyperparameter		Train, AUC	Test, AUC
SVM	cost	4.720295	0.647	0.624
	kernel	polynomial	(0.624, 0.671)	(0.587, 0.660)
	gamma	8.397468		
	type	C-classification		
	degree	1		
DT	xval	0	0.668	0.636
	cp	0.01103474	(0.646, 0.691)	(0.600, 0.671)
	minsplit	1		
	maxdepth	9		
	minbucket	6		
RF	num.threads	1	0.865	0.656
	num.trees	822	(0.850, 0.880)	(0.620, 0.691)
	mtry	1		
	min.node.size	4		
	max.depth	7		
KNN	k	15	0.737	0.607
	distance	1.807957	(0.716, 0.757)	(0.570, 0.643)
	kernel	rectangular		
XGBoost	nrounds	800	0.706	0.642
	nthread	1	(0.684, 0.728)	(0.606, 0.677)
	verbose	0		
	eta	1		
	gamma	5		
	max_depth	1		
	subsample	0.9921252		
	eval_metric	mlogloss		
GBM	n.cores	1	0.707	0.657
	n.trees	501	(0.685, 0.729)	(0.622, 0.693)
	n.minobsinnode	5		
	interaction.depth	1		
LR	-	-	0.674<br>(0.651, 0.696)	0.657<br>(0.621, 0.692)

Table S9. Summary of missingness patterns and descriptive statistics of all variables (N=3626, including normal, MCI and dementia)

Variable	Total	n	Missing Count	Missing Rate	mean	sd	median	trimmed	mad	min	max	range	skew	kurtosis	se
MoCA	3626	3626	0	0	0.58	0.70	0	0.47	0	0	2	2	0.80	-0.61	0.01
Age	3626	3626	0	0	58.30	9.89	59	58.41	10.38	35	86	51	-0.06	-0.55	0.16
Sex	3626	3626	0	0	1.66	0.47	2	1.70	0	1	2	1	-0.67	-1.55	0.01
Family members	3626	3601	25	0.69	3.07	1.38	3	2.96	1.48	1	9	8	0.72	-0.30	0.02
Education	3626	3623	3	0.08	0.73	0.70	1	0.67	1.48	0	2	2	0.41	-0.91	0.01
Marital status	3626	3617	9	0.25	0.13	0.39	0	0.02	0	0	2	2	3.05	9.07	0.01
Ethnic	3626	3621	5	0.14	0.39	0.56	0	0.31	0	0	2	2	1.13	0.29	0.01
Income	3626	3616	10	0.28	0.31	0.46	0	0.26	0	0	1	1	0.83	-1.31	0.01
Diabetes	3626	3626	0	0	0.07	0.25	0	0	0	0	1	1	3.44	9.81	0.00
Hypertension	3626	3626	0	0	0.25	0.43	0	0.19	0	0	1	1	1.16	-0.67	0.01
CHD	3626	3626	0	0	0.13	0.34	0	0.04	0	0	1	1	2.19	2.78	0.01
High	3626	3624	2	0.06	160.38	7.90	160	160.19	7.41	132	189	57	0.22	-0.12	0.13
Weight	3626	3624	2	0.06	63.53	11.50	63	63.08	11.86	33	120	87	0.45	0.49	0.19
BMI	3626	3624	2	0.06	24.64	3.72	24.52	24.53	3.70	14.53	39.89	25.36	0.36	0.37	0.06
Waist circumference	3626	3624	2	0.06	83.74	10.03	84	83.74	10.38	40	123.50	83.50	0.02	0.16	0.17
Hip circumference	3626	3624	2	0.06	94.72	7.79	95	94.68	7.41	41	188	147	0.16	8.30	0.13
WHR	3626	3624	2	0.06	0.88	0.07	0.88	0.88	0.06	0.40	1.88	1.49	1.89	24.96	0.00
SBP	3626	3618	8	0.22	132.95	20.89	131	131.49	20.26	79.33	254.67	175.33	0.77	1.10	0.35
DBP	3626	3618	8	0.22	80.23	11.02	79.67	79.87	11.37	47.67	135.67	88	0.36	0.24	0.18
Heart rate	3626	3618	8	0.22	75.69	11.31	74.33	75.08	10.38	41	159.33	118.33	0.70	1.58	0.19
Daily salt intake	3626	3454	172	4.74	19.91	11.40	16.67	18.52	8.24	0.56	125	124.44	2.32	10.17	0.19

Variable	Total	n	Missing Count	Missing Rate	mean	sd	median	trimmed	mad	min	max	range	skew	kurtosis	se
Daily oil intake	3626	3512	114	3.14	151.50	77.21	166.67	143.95	61.78	2.47	1666.67	1664.20	3.53	48.26	1.30
Weight management	3626	3626	0	0	0.05	0.22	0	0	0	0	1	1	4.16	15.30	0.00
Current smoker	3626	3625	1	0.03	1.64	0.48	2	1.68	0	1	2	1	-0.61	-1.63	0.01
Current drinker	3626	3622	4	0.11	0.26	0.44	0	0.20	0	0	1	1	1.08	-0.84	0.01
Home passive smoker	3626	3611	15	0.41	1.64	0.48	2	1.68	0	1	2	1	-0.59	-1.65	0.01
Tea	3626	3624	2	0.06	1.46	0.50	1	1.45	0	1	2	1	0.16	-1.97	0.01
Daily intake of fruit and vegetables	3626	3615	11	0.3	2.48	0.68	2	2.45	1.48	1	4	3	0.27	-0.20	0.01
Weekly intake of fish	3626	3602	24	0.66	1.40	0.58	1	1.33	0	1	4	3	1.14	0.59	0.01
Weekly intake of sugar	3626	3605	21	0.58	1.34	0.55	1	1.26	0	1	4	3	1.41	1.43	0.01
Weekly intake of beverage	3626	3543	83	2.29	1.05	0.25	1	1	0	1	4	3	6.02	44.05	0.00
Weekly intake of Cereal	3626	3611	15	0.41	2.69	1.12	3	2.74	1.48	1	4	3	-0.23	-1.33	0.02
Physical labour level	3626	3591	35	0.97	0.71	0.46	1	0.76	0	0	2	2	-0.78	-0.98	0.01
Moderate intensity exercise	3626	3562	64	1.77	0.40	0.77	0	0.25	0	0	2	2	1.48	0.34	0.01
TMAO	3626	1452	2174	59.96	6.14	9.49	3.96	4.69	3.34	0.00	252.92	252.92	13.57	319.03	0.25
Choline	3626	1452	2174	59.96	201.06	126.65	179.14	187.06	84.58	6.08	2489.02	2482.93	5.10	74.74	3.32
Betaine	3626	1452	2174	59.96	115.40	68.03	100.53	107.53	47.35	2.74	540.66	537.92	1.78	5.53	1.79
Carnitine	3626	1452	2174	59.96	56.42	26.55	50.28	53.77	18.95	3.66	207.28	203.62	1.16	2.29	0.70
ALT	3626	3606	20	0.55	0.04	0.20	0	0	0	0	1	1	4.61	19.23	0.00
GLU	3626	3606	20	0.55	0.22	0.42	0	0.16	0	0	1	1	1.32	-0.26	0.01
TCHOL	3626	3606	20	0.55	0.46	0.50	0	0.45	0	0	1	1	0.17	-1.97	0.01
HDL	3626	3606	20	0.55	0.29	0.45	0	0.24	0	0	1	1	0.93	-1.13	0.01
LDL	3626	3606	20	0.55	0.43	0.50	0	0.41	0	0	1	1	0.28	-1.92	0.01

Variable	Total	n	Missing Count	Missing Rate	mean	sd	median	trimmed	mad	min	max	range	skew	kurtosis	se
Creatinine	3626	3626	0	0	0.03	0.16	0	0	0	0	1	1	5.83	32.01	0.00
Uric acid	3626	3626	0	0	0.06	0.24	0	0	0	0	1	1	3.64	11.25	0.00
Subjective sleep quality	3626	3626	0	0	0.91	0.82	1	0.83	1.48	0	3	3	0.66	-0.11	0.01
Sleep onset latency	3626	3626	0	0	0.91	1.03	1	0.76	1.48	0	3	3	0.81	-0.58	0.02
Sleep duration	3626	3626	0	0	1.22	0.98	1	1.15	1.48	0	3	3	0.35	-0.91	0.02
Sleep efficiency	3626	3626	0	0	0.30	0.75	0	0.09	0	0	3	3	2.56	5.57	0.01
Sleep disturbance	3626	3626	0	0	1.08	0.52	1	1.09	0	0	3	3	0.24	1.06	0.01
Hypnotic drug use	3626	3626	0	0	0.05	0.35	0	0	0	0	3	3	6.84	47.66	0.01
Daytime disfunction	3626	3626	0	0	1.04	1.02	1	0.92	1.48	0	3	3	0.53	-0.96	0.02
PSQI	3626	3626	0	0	0.39	0.49	0	0.37	0	0	1	1	0.43	-1.81	0.01

MoCA Montreal cognitive assessment, *CHD* Coronary heart disease, *BMI* body mass index, *WHR* waist-hip ratio, *SBP* systolic blood pressure, *DBP* diastolic blood pressure, *TMAO* trimethylamine N-oxide, *ALT* alanine aminotransferase, *GLU* fasting blood glucose, *TCHOL* total cholesterol, *HDL* high-density lipoprotein cholesterol, *LDL* low-density lipoprotein cholesterol (LDL-C), *PSQI* Pittsburgh sleep quality index.

Table S10. Baseline characteristics by TMAO (N=3173)

Characteristics	TMAO N=1294	Without TMAO N=1879	*p*-value
Age, ≥ 60y	620 (47.9%)	884 (47.0%)	0.580
Male, n (%)	411 (31.8%)	660 (35.1%)	0.051
Education level, n (%)			0.210
Primary school or below	489 (37.8%)	733 (39.0%)	
Junior high school	585 (45.2%)	870 (46.3%)	
Tertiary high school or higher	220 (17.0%)	276 (14.7%)	
Marital status, n (%)			0.119
Independent	16 (1.2%)	42 (2.2%)	
Married	1177 (91.0%)	1693 (90.1%)	
Widowhood	101 (7.8%)	144 (7.7%)	
Ethnicity, n (%)			0.068
Han	830 (64.1%)	1243 (66.2%)	
Mongolian	417 (32.2%)	546 (29.1%)	
Others	47 (3.6%)	90 (4.8%)	
Income, ＜10,000 Yuan, n (%)	916 (70.8%)	1295 (68.9%)	0.271
Family members, person (N=3151)	3.04 ± 1.37	3.11 ± 1.36	0.104
Diabetes, n (%)	90 (7.0%)	107 (5.7%)	0.155
Hypertension, n (%)	360 (27.8%)	548 (29.2%)	0.434
CHD, n (%)	188 (14.5%)	306 (16.3%)	0.218
High, cm (N=3171)	160.47 ± 7.56	160.82 ± 7.96	0.240
Weight, kg (N=3171)	63.94 ± 11.11	64.06 ± 11.82	0.966
BMI, kg/m^2 (N=3171)	24.79 ± 3.70	24.70 ± 3.75	0.599
Waist circumference, cm (N=3171)	84.15 ± 10.00	83.66 ± 10.05	0.166
Hip circumference, cm (N=3171)	95.06 ± 8.13	94.89 ± 7.60	0.615
WHR (N=3171)	0.89 ± 0.08	0.88 ± 0.07	0.073
SBP, mmHg (N=3166)	132.79 ± 20.07	131.91 ± 21.01	0.104
DBP, mmHg (N=3166)	80.09 ± 10.60	80.50 ± 11.37	0.617
Heart rate, bpm (N=3166)	75.45 ± 11.18	75.79 ± 11.20	0.318
Daily salt intake, g (N=3037)	20.30 ± 12.14	19.39 ± 10.17	0.383
Daily oil intake, ml (N=3078)	147.48 ± 66.88	152.51 ± 80.81	0.267
Weight management, n (%)	81 (6.3%)	95 (5.1%)	0.169
Current smoker, n (%)	427 (33.0%)	671 (35.7%)	0.120
Current drinker, n (%)	268 (20.7%)	441 (23.5%)	0.065

Home passive smoker, n (%)	466 (36.0%)	670 (35.7%)	0.851
Tea, n(%)	698 (53.9%)	1033 (55.0%)	0.586
Daily intake of fruit and vegetables, n (%)			0.478
< 250g	52 (4.0%)	75 (4.0%)	
250g-500g	625 (48.3%)	925 (49.2%)	
> 500g	673 (47.7%)	879 (46.8%)	
Weekly intake of fish, n (%)			0.098
< 100g	823 (63.6%)	1170 (62.3%)	
100g-250g	414 (32.0%)	616 (32.8%)	
> 250g	57 (4.5%)	93 (4.9%)	
Weekly intake of sugar, n (%)			0.510
< 50g	867 (67.0%)	1222 (65.0%)	
50g-100g	380 (29.4%)	592 (31.5%)	
> 100g	47 (3.6%)	65 (3.4%)	
Weekly intake of beverage, ＜ 500ml, n (%)	1236 (95.5%)	1789 (95.2%)	0.134
Weekly intake of Cereal, n (%)			0.337
< 100g	247 (19.1%)	358 (19.1%)	
100g-250g	275 (21.3%)	434 (23.1%)	
250g-1kg	360 (27.8%)	473 (25.2%)	
> 1kg	412 (31.8%)	614 (32.6%)	
Physical labour level, n (%)			0.310
moderate	935 (72.3%)	1367 (69.3%)	
low	4 (0.3%)	13 (0.7%)	
high	355 (27.4%)	499 (25.3%)	
Moderate intensity exercise, weekly times, n(%)			0.218
< 3	992 (76.7%)	1402 (74.6%)	
3-4	74 (5.7%)	100 (5.3%)	
> 4	228 (17.6%)	377 (20.1%)	
Choline, μmol/L (N=1294)	195.00 ± 108.26	-	-
Betaine, μmol/L (N=1294)	112.58 ± 63.27	-	-
Carnitine, μmol/L (N=1294)	55.95 ± 25.69	-	-
ALT, abnormal, n (%)	57 (4.4%)	87 (4.6%)	0.795
GLU, abnormal, n (%)	304 (23.5%)	404 (21.5%)	0.193
TCHOL, abnormal, n (%)	610 (47.1%)	864 (46.0%)	0.544
HDL, abnormal, n (%)	368 (28.4%)	562 (29.9%)	0.383
LDL, abnormal, n (%)	581 (44.9%)	881 (46.9%)	0.286
Creatinine, abnormal, n (%)	36 (2.8%)	47 (2.5%)	0.652
Uric acid, abnormal, n (%)	85 (6.6%)	117 (6.2%)	0.712
Subjective sleep quality, n (%)			0.627
Very good (0 score)	451 (34.9%)	668 (35.6%)	
Fairly good (1 score)	569 (44.0%)	839 (44.7%)	
Fairly bad (2 score)	221 (17.1%)	289 (15.4%)	
Very bad (3 score)	53 (4.1%)	83 (4.4%)	
Sleep onset latency, n (%)			0.507

Fast (0 score)	592 (45.7%)	908 (48.3%)	
Fairly slow (1 score)	364 (28.1%)	503 (26.8%)	
Slow (2 score)	190 (14.7%)	272 (14.5%)	
Very slow (3 score)	148 (11.4%)	196 (10.4%)	
Sleep duration, n (%)			0.245
> 7 h (0 score)	322 (24.9%)	510 (27.1%)	
6-7 h (1 score)	496 (38.3%)	687 (36.6%)	
5-6 h (2 score)	328 (25.3%)	443 (23.6%)	
< 5h (3 score)	148 (11.4%)	239 (12.7%)	
Sleep efficiency, n (%)			0.497
> 85% (0 score)	1094 (84.5%)	1564 (83.2%)	
75-84% (1 score)	100 (7.7%)	159 (8.5%)	
65-74% (2 score)	57 (4.4%)	77 (4.1%)	
< 65% (3 score)	43 (3.3%)	79 (4.2%)	
Sleep disturbance, n (%)			0.060
Not at all (0 score)	108 (8.3%)	201 (10.7%)	
1-9 (1 score)	942 (72.8%)	1337 (71.7%)	
10-18 (2 score)	239 (18.5%)	339 (18.2%)	
19-28 (3 score)	5 (0.4%)	2 (0.1%)	
Hypnotic drug use, n (%)			0.889
Not during the past month (0 score)	1264 (97.7%)	1828 (97.3%)	
Less than once a week (1 score)	8 (0.6%)	12 (0.6%)	
Once or twice a week (2 score)	14 (1.1%)	26 (1.4%)	
Three or more times a week (3 score)	8 (0.6%)	13 (0.7%)	
Daytime disfunction, n (%)			0.117
Not at all (0 score)	454 (35.1%)	738 (39.3%)	
1-2 times (1 score)	374 (28.9%)	503 (26.8%)	
3-4 times (2 score)	322 (24.9%)	447 (23.8%)	
5-6 times (3 score)	144 (11.1%)	191 (10.2%)	
PSQI, sleep disorder, n (%)	526 (40.6%)	704 (37.5%)	0.075

Data are mean (SD), median [interquartile range], or n (%). P value of continuous variables are calculated by Student's t-test or Mann–Whitney U test, and categorical variables are calculated by $\chi2$ test or Wilcoxon rank-sum test.

TMAO trimethylamine N-oxide, CHD Coronary heart disease, BMI body mass index, WHR waist-hip ratio, SBP systolic blood pressure, DBP diastolic blood pressure, ALT alanine aminotransferase, GLU fasting blood glucose, TCHOL total cholesterol, HDL high-density lipoprotein cholesterol, LDL low-density lipoprotein cholesterol (LDL-C), PSQI Pittsburgh sleep quality index.
